# Using a Relative Quantitative Proteomic Method to Identify Differentially Abundant Proteins in *Brucella melitensis* Biovar 3 and *Brucella melitensis* M5-90

**DOI:** 10.3389/fimmu.2022.929040

**Published:** 2022-07-19

**Authors:** Huan Zhang, Yueli Wang, Yifan Wang, Xiaoyu Deng, Taiwang Ji, Zhongchen Ma, Ningning Yang, Mingguo Xu, Honghuan Li, Jihai Yi, Yong Wang, Yuanzhi Wang, Jinliang Sheng, Zhen Wang, Chuangfu Chen

**Affiliations:** ^1^ School of Animal Science and Technology, Shihezi University, Shihezi City, China; ^2^ State Key Laboratory of Agricultural Microbiology, College of Veterinary Medicine Huazhong Agricultural University, Wuhan, China; ^3^ School of Medicine, Shihezi University, Shihezi City, China

**Keywords:** Brucellosis, *Brucella melitensis* M5-90, *Brucella melitensis* biovar 3, proteomics, vaccine

## Abstract

Brucellosis, caused by *Brucella* spp., is one of the most widespread bacterial zoonoses worldwide. Vaccination is still considered the best way to control brucellosis. An investigation into the differential proteome expression patterns of wild and vaccine strains may help researchers and clinicians differentiate between the strains to diagnose and better understand the mechanism(s) underlying differences in virulence. In the present study, a mass spectrometry-based, label-free relative quantitative proteomics approach was used to investigate the proteins expressed by the wild strain, *B. melitensis* biovar 3 and compare it with those expressed by *B. melitensis* M5-90. The higher level of virulence for *B. melitensis* biovar 3 compared to *B. melitensis* M5-90 was validated *in vitro* and *in vivo*. A total of 2133 proteins, encompassing 68% of the theoretical proteome, were identified and quantified by proteomic analysis, resulting in broad coverage of the *B. melitensis* proteome. A total of 147 proteins were identified as differentially expressed (DE) between these two strains. In addition, 9 proteins and 30 proteins were identified as unique to *B. melitensis* M5-90 and *B. melitensis* biovar 3, respectively. Pathway analysis revealed that the majority of the DE proteins were involved in iron uptake, quorum sensing, pyrimidine metabolism, glycine betaine biosynthetic and metabolic processes, thiamine-containing compound metabolism and ABC transporters. The expression of BtpA and VjbR proteins (two well-known virulence factors) in *B. melitensis* biovar 3 was 8-fold and 2-fold higher than in *B. melitensis* M5-90. In summary, our results identified many unique proteins that could be selected as candidate markers for differentiating vaccinated animals from animals with wild-type infections. BtpA and VjbR proteins might be responsible for the residual virulence of *B. melitensis* M5-90, while ABC transporters and thiamine metabolism associated proteins may be newly identified *Brucella* virulence factors. All of the identified DE proteins provide valuable information for the development of vaccines and the discovery of novel therapeutic targets.

## Introduction

Identification and characterization of virulence factors can provide a roadmap for the development of diagnostic methods, therapeutic drugs, and vaccines (1). However, it is difficult to identify disease-related proteins and mechanisms in pathogenic species due to the multifactorial nature of pathogenicity. Comparisons between virulent and attenuated strains of a pathogenic species are expected to provide valuable information on determinants of biology and pathogenicity.

Brucellosis, caused by *Brucella Spp*., is a prominent zoonosis in the Mediterranean region, Middle East, and Central Asia (2) that causes severe health problems in humans, as well as economic losses. Globally, more than 500,000 cases of human brucellosis occur each year (3). In China, 47,245 human cases were reported in 2020 (https://www.chinacdc.cn), and the annual economic losses due to this disease are estimated at 16,386,500 yuan (2,589,067 USD) (4). The incidence of brucellosis in humans and animals is much higher in areas where the economy is dependent on animal production.

The Xinjiang Uygur Autonomous Region (XUAR) is located in the northwest of China. Animal husbandry has played a key role in the development of the economy in the XUAR. In 2020, beef production (439,900 tons) and mutton production (569,800 tons) in Xinjiang accounted for 6.5% and 11.6%, respectively, of the total beef and mutton production in China as reported by the National Bureau of Statistics (http://www.stats.gov.cn). However, Xinjiang is also an endemic area for brucellosis with high incidences in 2015 in cattle (2.05%) and sheep/goats (3.96%) (Data from the Center for Animal Disease Control and Prevention of Xinjiang). Brucellosis morbidity, in both humans and animals, is increasing, especially in remote areas. Recently, several studies have reported that *Brucella melitensis* (*B. melitensis*) biovar 3 is the predominant wild strain in Xinjiang. This strain has been isolated from milk, aborted sheep fetuses, human blood, and Asian badgers (5–10).

Vaccination is the most efficient measure for controlling brucellosis. Currently, in Xinjiang, live attenuated *B. melitensis* M5-90 is widely used to inoculate sheep and goats for the prevention of brucellosis as it confers good protective immunity for animals. However, the residual virulence of *B. melitensis* M5-90 is an issue, and the vaccine can cause abortion in gestational animals and contaminate milk during lactation (11, 12). In addition, both the *Brucella* wild strain and *B. melitensis* M5-90 have the O-polysaccharide (OPS), which can interfere with serological diagnostic test results; thus, differentiating infected from vaccinated animals (DIVA) remains a huge challenge (11, 13, 14). To overcome these hurdles, many studies have investigated bacterial biology and pathogenic mechanisms of *B. melitensis* Rev.1 and *B. melitensis* 16M (15–19). However, few studies have examined bacterial biology and pathogenic mechanisms of *B. melitensis* M5-90 and *B. melitensis* biovar 3.

Previous studies have suggested that the differential expression of proteins contributes to pathogenicity or virulence level between different strains (20, 21). The identification of differences in protein expression and/or protein abundance using proteomics may provide deep insights into biology and virulence. In the current study, label-free proteomics is used to identify differences in protein expression between the *B. melitensis* M5-90 vaccine strain and the *B. melitensis* biovar 3 wild strain. The aims of this study are as follows: 1) to compare and screen the differentially expressed proteins as candidate targets for DIVA; and 2) to understand the differences in virulence according to the differential expression of proteins between the two strains and search for potential proteins that may contribute to the remaining virulence in *B. melitensis* M5-90.

## Materials and Methods

### Bacterial Strains, Phylogenetic Analysis and Culture Conditions

The *B. melitensis* vaccine strain M5-90 was donated by the Xinjiang Tiankang Animal Biotechnology Co., Ltd. (China). *B. melitensis* biovar 3 was isolated from milk or aborted sheep fetuses in which the species and biotype had been identified in previous work (9). Phylogenetic trees of the isolate were constructed according to the 731 bp sequence of the IS*711* repetitive element. The neighbor-joining (NJ) method and maximum-likelihood algorithms were used to calculated the sequence distance and were done with Molecular Evolutionary Genetics Analysis (MEGA) 7 software (22). *B. melitensis* M5-90 and *B. melitensis* biovar 3 (1 mL) were inoculated into 200 mL tryptic soy broth (TSB, Biolife) and cultured for 3 d at 37°C and 150 rpm. Bacterial cells were collected by centrifugation at 12,000 x g for 5 min at 4°C, washed with sterile ice-cold 0.01 M phosphate-buffered saline (PBS, Solarbio LIFE SCIENCES, Beijing, China), killed at 85°C for 1 h, and stored at −80°C.

### Macrophage Infection With *B. melitensis* M5-90 and *B. melitensis* Biovar 3

Murine macrophage RAW264.7 cell lines (ATCC, TIB-71) were plated on 6-well plates at 5 × 10^5^ cells/mL in 2 mL Dulbecco’s modified Eagle’s medium (DMEM, Gibco Life Technologies, Rockville, MD, USA) supplemented with 10% fetal serum and cultured for 24 h at 5% CO_2_ 37°C. Then, the cells were infected with *B. melitensis* M5-90 or *B. melitensis* biovar 3 at a multiplicity of infection (MOI) of 100. At indicated time points post infection, cells were washed three times with sterile PBS to remove extracellular bacteria and 1 mL of cell lysis buffer (PBS + 0.1% Triton X-100) per well was then added. Live bacteria were counted and calculated by plating on tryptic soy agar (TSA, Biolife).

### Balb/c Mouse Experimental Model of Brucellosis

Six to eight-week-old Balb/c female mice were purchased from the Experimental Animal Center of the Academy of Military Medical Science (Beijing, China). Animals were maintained in a biosafety level 3 laboratory and were supplied with enough food and water. All mice were acclimatized for 1 week before experimentation. Mice were inoculated, *via* intraperitoneal injection, with 10^6^ CFU/0.2 mL *B. melitensis* M5-90 or *B. melitensis* biovar 3 diluted in PBS; 0.2 mL PBS was used as the negative control. Mice were euthanized at indicated time points post-infection according to institutional animal care guidelines. Spleens were isolated aseptically from individual mice and then weighed and homogenized in a tissue grinder for 10 min. The tissue suspensions were serially diluted and plated on TSA for the determination of colony forming units (CFU).

### Protein Extraction and Trypsin Digestion

The inactivated *Brucella* strain was resuspended in lysis buffer (8M urea, 1% Protease Inhibitor Cocktail) and sonicated three times on ice using a high intensity ultrasonic processor (Scientz, Ningbo, China). The remaining debris was removed by centrifugation at 12,000 g at 4°C for 10 min. The supernatant was collected and extracted proteins were quantified using a Pierce Microplate BCA Protein Assay Kit (Thermo, Waltham, MA, USA) according to the manufacturer’s instructions. The concentrations of extracted proteins are listed in **Table S1**.

A 300-μg protein sample was used for digestion, and the protein solution was reduced with 5 mM dithiothreitol for 30 min at 56°C and alkylated with 11 mM iodoacetamide for 15 min in the dark at room temperature. The protein sample was diluted by adding 100 mM triethylammonium bicarbonate (Thermo, USA) so that the urea concentration was less than 2 M. Finally, trypsin was added at a 1:50 trypsin-to-protein mass ratio for initial overnight digestion and a 1:100 trypsin-to-protein mass ratio was added for a second 4 h-digestion.

### LC-MS/MS Analysis

A total of 5 μg of tryptic peptides was dissolved in 0.1% formic acid (solvent A) and directly loaded onto a homemade reversed-phase analytical column (15-cm length, 75 μm i.d.). The gradient was comprised of an increase from 6% to 23% solvent B (0.1% formic acid in 98% acetonitrile) over 26 min, 23% to 35% over 8 min, climbed to 80% in 3 min, and then held at 80% for the final 3 min. All steps were done at a constant flow rate of 300 nL/min in a nanoElute UHPLC system (Bruker Daltonics).

In total, 0.2 μg of peptides was subjected to capillary electrophoresis followed by timsTOF Pro (Bruker Daltonics) mass spectrometry. The electrospray voltage applied was 1.4 kV. Precursors and fragments were identified and analyzed using the TOF detector, with the MS/MS scan range set at 100 to 1700 m/z. The timsTOF Pro was operated in the parallel accumulation serial fragmentation (PASEF) mode. Precursors with charge states from 0 to 5 were selected for fragmentation, and 10 PASEF-MS/MS scans were acquired each cycle. The dynamic exclusion was set at 24 s.

### Database Search

The resulting MS/MS data were processed using Maxquant (v1.6.6.0) and searched against the Uniprot database (*Brucella melitensis*, 3138 entries. UniprotKB: UP000013102) concatenated with the reverse decoy database. Trypsin/P was specified as a cleavage enzyme and up to 2 missed cleavages. The mass tolerance for precursor ions was set at 70 ppm for the first search and 70 ppm for the main search, and the mass tolerance for fragment ions was set at 0.04 Da. Carbamidomethyl on Cys was specified as fixed modification and oxidation on Met and acetylation on the protein N-terminus were specified as variable modifications. The false discovery rate (FDR) was adjusted to < 1%, and the minimum score for modified peptides was set to > 40.

### Bioinformatics Data Analysis

Protein’s Gene Ontology (GO) function and the protein domain function annotation were completed using the InterProScan software (v.5.14-53.0), according to the protein sequence alignment method. Then, the classification of proteins was finished by GO annotation based on three categories: biological process, cellular component, and molecular function. Additionally, annotation of the protein pathway was completed using the Kyoto Encyclopedia of Genes and Genomes (KEGG) database; Wolfpsort software (v.0.2) was used to predict subcellular localization. GO and the KEGG databases were used to perform functional enrichment analysis. The corresponding functions and pathways with *P* < 0.05 (two-tailed Fisher’s exact test) were considered significant.

### RNA Extraction and qRT-PCR Analysis

Total RNA was isolated from RAW264.7 cells (infected with *B. melitensis* Y3 or *B. melitensis* M5-90) at different points in time using an RNeasy Kit (CWBIO, Beijing, China). DNA was eliminated using a TURBO DNA-free DNAse (Ambio). The concentration and quality were assessed *via* a Nanodrop 2000 (Thermo, USA). RNA samples were subjected to reverse transcription using a HiFiScript cDNA Kit (CWBIO, Beijing, China) according to the manufacturer’s instructions. Real-time PCR analysis was performed using ThermoFisher QuantStudio 3 RT PCR-well Q3 (Thermo Fisher, USA) with SYBR (CWBIO, Beijing, China). The primers for target genes were as follows: 16 S forward, 5′-ACTAAGGGCGAGGGTTGC-3′; 16 S reverse, 5′-CACTGGACCATTACTGACGC-3′; BtpA forward, 5′-GCCCGCAAGAGAATTAGATGGACTG-3′; BtpA reverse, 5′-GAGGGACTGAAACGCCGAACTTC-3′; vjbR forward, 5′-CCGCTACGTAACGCATACCTATCG-3′; vjbR reverse, 5′-CAGGTAGCAGGCAGCGTCATAAG-3′. The reaction conditions were as follows: initial denaturation at 95°C for 5 min followed by 45 cycles of 95°C for 30 s and annealing at 57°C or 60°C for 30 s. Fold changes of each gene were calculated using the 2^-ΔΔCT^ method and the mRNA levels were normalized according to 16 S ribosomal RNA expression.

### Ethics Statement

This research was approved by the Animal Care and Use Committee of Shihezi University. All animals used in this study were treated humanely and in accordance with institutional animal care guidelines.

### Statistical Analysis

All experiments (except proteomics) were repeated at least three times. The data are presented as mean ± standard deviation. Graphs and data analysis were performed with R (version 4.0.5), using the “ggplot2” package and unpaired Student’s test. Asterisks in the figures represent statistical significance (* *P* < 0.05, ** *P* < 0.01, *** *P* < 0.005, ns not significant).

## Results

### Survival of *B. melitensis* M5-90 (M5) and *B. melitensis* biovar 3 (Y3) *In Vitro* and *In Vivo*

**Figure 1:**
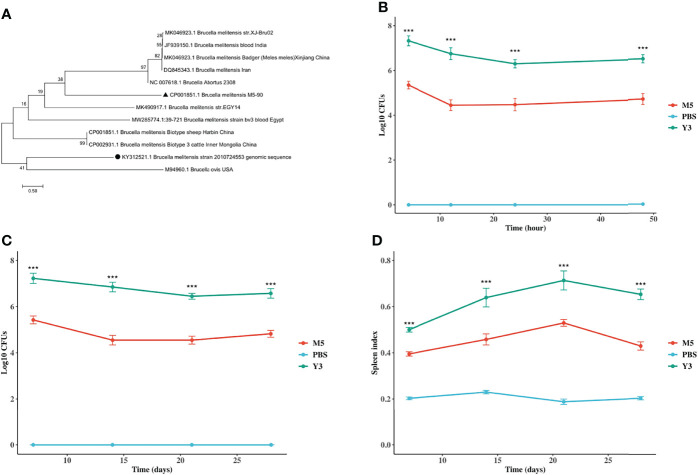
Investigation of the phylogeny of B. *melitensis* biovar 3 and B. *melitensis* M5-90, and identification of survivability of B. *melitensis* biovar 3 and B. *melitensis* M5-90 *in vitro* and *in vivo*. **(A)** Phylogenetic tree of the IS*711* concatenated sequence of B. *melitensis* biovar 3 (**●**) isolated from milk or aborted sheep fetuses in a previous study and reference sequences from B. *melitensis* retrieved from the GenBank database. The black triangle (▲) represents *Brucella melitensis* M5-90. The tree was constructed according to the neighbor-joining (NJ; 500 bootstrap replicates) and maximum-likelihood (ML, 1000 bootstrap replicates) analyses using MEGA7. The scale bar represents the inferred substitutions per nucleotide site; **(B)** RAW264.7 cell lines were infected with B. *melitensis* biovar 3 or B. *melitensis* M5-90 for 4, 12, 24, 48h. The CFU was determined on TSA at each time point; **(C)** The Balb/c mice were infected with B. *melitensis* biovar 3 or B. *melitensis* M5-90 and PBS (negative control) with four mice in each group. At 7, 14, 21, 28 days post-infection, the spleen was isolated and individual spleens were assessed for colonization and weight index **(D)**. Splenic weight was calculated as organ weight (in grams) per gram of mouse body weight.

A phylogenetic tree was constructed to investigate the phylogenetic relationship of the Y3 isolate. The tree shows that the isolate imperfectly matched the *B. melitensi*s biotype 3 isolated from sheep and cattle in Harbin and Inner Mongolia, but matched the *Brucella* ovis. (**Figure 1A**). It might be a new *B. melitensis* biovar 3 strain compared with those Y3 strains isolated from other provinces in China. To compare the survival and/or replication ability of Y3 and M5, RAW264.7 macrophages, and Balb/c mice were infected with Y3 or M5. From 4 h to 48 h, the survivability of Y3 was 2-log higher than that of M5 (**Figure 1B**), and the number of bacteria in the Y3 group was 2-log higher than in the M5 group (**Figure 1C**) at all indicated time points post-infection *in vivo*. In addition, the spleen index indicated that the degree of splenomegaly in the Y3 group was 0.2- and 0.3-fold higher than in the M5 group (**Figure 1D**). Overall, these results suggest that the survivability and pro-inflammatory ability of the Y3 wild strain are stronger than those of the M5 vaccine strain.

### Protein Identification in the M5 and Y3 Strains

**Figure 2:**
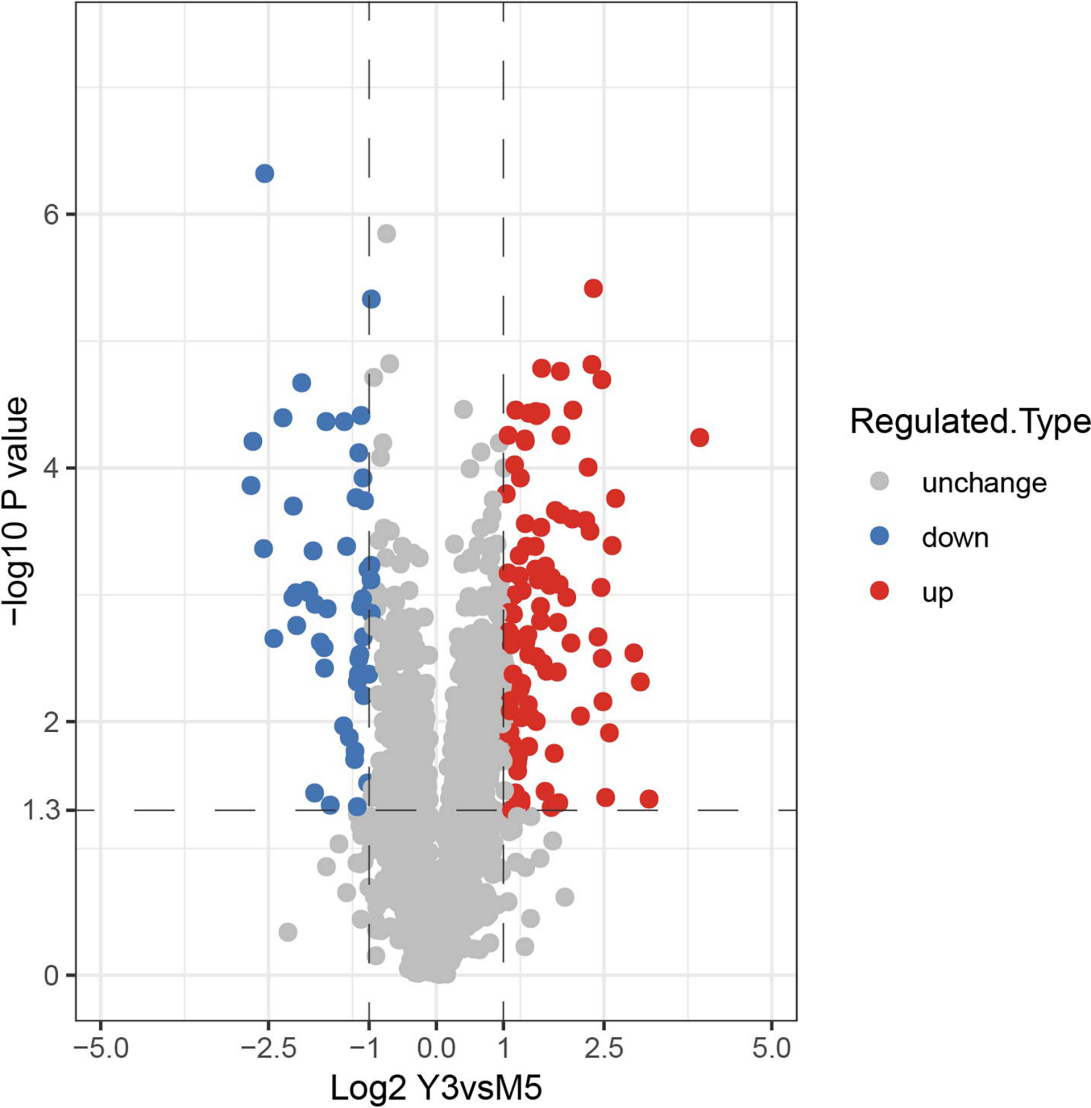
Identification of DE proteins of *B. melitensis* biovar 3 and *B. melitensis* M5-90. The volcano plot also shows the DE proteins of *B. melitensis* biovar 3 and *B. melitensis* M5-90; red points represent hDE proteins and blue points represent lDE proteins. The protein dataset in this study is classified according to the relative abundance of the protein entries, calculated on the inter-sample (i.e. Y3 vs M5) basis.

To investigate the differentially expressed (DE) proteins in the M5 and Y3 strains. Peptides of these two samples were processed and analyzed using the LC-MS. A total of 269,817 second-order spectra were identified by mass spectrum analysis, of which the number of available mass spectra was 133,347 (49.4%). A total of 16,911 peptides were identified by mass spectrum analysis, of which the number of specific peptides was 16,111 (**Figure S1A**). A total of 2,133 proteins were identified, which represents approximately 68% (2,133/3,138) coverage of the whole *B. melitensis* proteome and the number of quantifiable proteins was 1,766 (**Figure S1A**). In addition, 2,094 proteins were identified as shared between M5 and Y3; however, 9 proteins and 30 proteins were identified as unique to M5 and Y3, respectively (**Figure S1B**). Detailed unique proteins are listed in **Table S2**. The label-free quantification (LFQ) intensity of protein in each sample was obtained by LFQ calculation and the value of relative quantification was determined based on the different LFQ intensities in each sample. Specifically, the expressions of different proteins were calculated in the M5 and Y3 groups, and the *P* values of proteins were calculated in these two groups. *P* values were determined by a two-tailed Fisher’s exact test. A fold change > 2 was considered as high differentially expressed (hDE) and < 1/2 was considered as low differentially (lDE) expressed. A total of 147 proteins were identified as differentially expressed, of which 97 proteins were considered hDE and 50 proteins were considered lDE (**Figures 2**, **S2**). The detailed DE proteins are shown in **Table S3**. A total of 2133 proteins were identified in the Y3 and M5 strain, of which 147 proteins were identified as DE proteins; another 9 proteins were found to be unique to M5, and 30 to Y3.

Two statistical methods were used to examine the quantitative repeatability of the processes performed on these two samples. The PCA plot indicated good homogeneity of the three replicates in each group (**Figure S3A**). The boxplot was used to evaluate the intensity distribution of the two samples. The smaller the relative standard deviation value (RSD), the better the repeatability (**Figure S3B**). Collectively, these results indicate that the data reproducibility was high for the two samples.

### Classification of GO Secondary Annotation of DE Proteins

**Figure 3:**
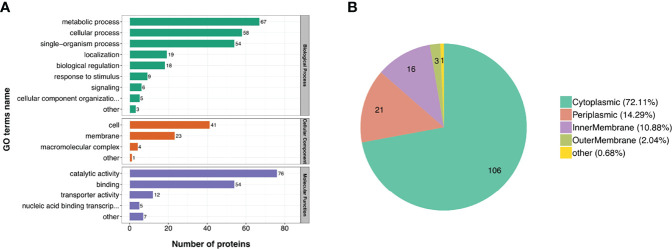
Classification of GO secondary annotation and subcellular location of DE proteins. **(A)** The biological function of DE proteins was characterized by biological process, cellular component, and molecular function; **(B)** The pie chart shows the proportion of subcellular location of DE proteins.

To investigate the biological function of the DE proteins, the DE proteins were annotated using GO. The DE proteins identified in Y3 and M5 were found to be principally involved in the metabolic processes, cellular processes and single-organism processes, according to biological process classification. Most of the DE proteins were found to have catalytic and binding activity based on the molecular function classification. The DE proteins were identified as cell and membrane components (**Figure 3A**). Of the lDE proteins, 19 proteins (29.7%, 19/64), 17 proteins (26.6%, 17/64), and 13 proteins (20.3%, 13/64) participated in metabolic processes, single-organism processes, and cellular processes, respectively (**Figure S4A**). Of the hDE proteins, 48 proteins (27.4%, 48/175), 37 proteins (21.1%, 37/175), and 45 proteins (25.7%, 45/175) participated in metabolic processes, single-organism processes, and cellular processes, respectively (**Figure S4B**). There were more hDE proteins than lDE proteins involved in cellular processes. Subcellular localization of the M5 and Y3 DE proteins was also analyzed. The Y3 and M5 DE proteins were mainly located in the cytoplasm (72.11%), periplasm (14.29%) and inner membrane (10.88%) (**Figure 3B**). The lDE proteins were principally located in the cytoplasm (64%) and periplasm (24%), whereas the hDE proteins were mainly located in the cytoplasm (76.29%) and inner membrane (12.37%) (**Figures S5A, B**). These results indicate most of the DE proteins participated in metabolic processes, cellular processes, and single-organism processes. They were identified as either cell component or membrane components. Most of the DE proteins were also found to have catalytic and binding activity.

### The COG/KOG Function Categorization of DE Proteins

The DE proteins were analyzed based on the Clusters of Orthologous Group of proteins (COG) database and Clusters of Orthologous Groups for eukaryotic complete genomes (KOG) database to further explore their functions. The DE proteins were mainly involved in amino acid transport and metabolism (**Figure S6**). Most of the lDE proteins participated in amino acid transport and metabolism, general function, as well as some unknown functions, while the hDE proteins were mainly involved in amino acid transport and metabolism. (**Figures S7A, B**). These results suggest that most of the DE proteins are involved in amino acid transport and metabolism.

### GO Functional Categorization of the DE Proteins

The functional categorization of the DE proteins was examined using InterProScan to determine whether the DE proteins had significant specific functional categorizations (v.5.14-53.0). The DE proteins were found to be primarily involved in glycine betaine biosynthetic and metabolic processes, thiamine-containing compound metabolic processes, and inorganic ion transmembrane transport, according to the biological process annotation (**Figure S8A**). The lDE proteins were principally involved in water-soluble vitamin biosynthetic processes, vitamin biosynthetic processes, and water-soluble vitamin metabolic processes (**Figure S9A**). The hDE proteins were primarily involved in ion transmembrane transport and DNA metabolic processes (**Figure S9B**). The DE proteins mainly belonged to the cell envelope, envelope, and external encapsulating structure (**Figure S8B**). The lDE proteins were found in the cell envelope, envelope, and external encapsulating structure (**Figure S9C**), while the hDE proteins were found in the cytosol or cytosolic part, based on cellular component enrichment analysis (**Figure S9D**). Additionally, the majority of DE proteins were attributed to metal cluster binding and iron-sulfur cluster binding, based on the molecular function enrichment analysis (**Figure S8C**). The lDE proteins were mainly involved in ammonia-lyase activity and carbon-nitrogen lyase activity (**Figure S9E**) and the majority of hDE proteins were involved in ion transmembrane transporter activity, ribonucleoside-diphosphate reductase activity, and DNA polymerase activity (**Figure S9F**). The directed acyclic graph (DAGs) depicts the relationships, or hierarchy, of GO classification (biological process, cellular component, and molecular function) (**Figures S10A-C**). Collectively, these results indicate these DE proteins are mainly involved in glycine betaine biosynthetic and metabolic processes, thiamine-containing compound metabolic processes, and inorganic ion transmembrane transport and that most of them belong to the cell envelope, envelope, and external encapsulating structure. The majority of DE proteins were attributed to metal cluster binding and iron-sulfur cluster binding.

### KEGG Pathway Enrichment Analysis of DE Proteins

To investigate the specific pathways in which DE proteins may be involved, the DE proteins were analyzed using KEGG database. The DE proteins were found to be primarily involved in ABC transporters, quorum sensing, and pyrimidine metabolism, according to KEGG database enrichment analysis (**Figure S11**). Among the ABC transporters, the sulfate/thiosulfate, general L-amino acid, branched-chain amino acid, oligopeptide and nickel transporters were specifically found to be over-expressed (**Figure S12**). In the quorum sensing system, the sensing protein Zur, which is associated with iron uptake, multidrug resistance, and detoxification was found to be highly expressed (**Figure S13**). Additionally, many processing proteins involved in late competence gene expression, cysteine protease synthesis (indirectly related with virulence), enterotoxin/phospholipase synthesis (indirectly related with virulence), degradation enzyme synthesis (indirectly related with necrotrophism) and lipopeptide (indirectly related with biofilm formation) were also over-expressed (**Figure S13**). Many key enzymes in pyrimidine metabolism were found to be highly expressed including enzymes involved in orotate/dihydro-orotate metabolism, uracil/uridine monophosphate (UMP) metabolism, cytidine diphosphate (CDP)/deoxyribonucleoside diphosphate (dCDP) metabolism, uridine diphosphate (UDP)/deoxy uridine diphosphate (dUDP) metabolism, and thymine/(R)-dihydro-thymine metabolism (**Figure S14**).

The lDE proteins were mainly attributed to thiamine metabolism, degradation of aromatic compounds, and benzoate degradation (**Figure S15A**), whereas the majority of hDE proteins were related to ABC transporters, quorum sensing, and pyrimidine metabolism (**Figure S15B**). Specifically, low expression of several essential enzymes related to thiamine metabolism and that participate in thiamine phosphate synthesis and 4-amino-5-hydroxymethyl-2-methylpynmidine synthesis were found. Many substances related to the degradation of aromatic compounds were significantly reduced including catechol, 4-methylcatechol, 1/2-methylnaphthalene and 2,3-dihydroxy-ethylbenzene. Three key enzymes related to benzoate degradation were down-regulated; specifically, those associated with catechol/2-hydroxymuconate-semialdehyde metabolism, 3-sulfocatechol/2-hydroxymuconate metabolism and (S)-3-hydroxy-butanoyl-CoA/acetoacetyl-CoA metabolism (**Figures S16-18**). Interestingly, the specific results of enrichment analysis for hDE proteins including ABC transporters, quorum sensing and pyrimidine metabolism were similar to the results for the DE proteins described above rather than the lDE proteins (**Figures S12-14**). Overall, these results demonstrate the biological process, cellular component, and molecular function differences between the Y3 wild strain and M5 vaccine strain. The stronger virulence level of the Y3 strain compared to the M5 strain might be related to the over-expression of cysteine protease and enterotoxin/phospholipase.

### Protein Domain Categorization of DE Proteins

The protein domain refers to the repeated components, with similar sequences, structures, and functions, in different protein molecules and is a unit of protein evolution. The domain is 25 to 500 amino acids in length. To further investigate the protein domain function of DE proteins, the DE proteins were analyzed using protein domain enrichment analysis. The majority of DE protein domains are primarily associated with the ABC transporter type 1 transmembrane domain, glycosyl transferase, nitro reductase, and the dihydroorotate dehydrogenase domain (**Figure S19**). Specifically, the lDE protein domains are principally associated with glycosyl transferase and the glyoxalase/bleomycin resistance protein (**Figure S20A**); the hDE protein domains are mainly associated with the ABC transporter type 1 transmembrane domain, the dihydroorotate dehydrogenase domain, ribonucleotide reductase, and the transcription regulator LuxR, C-terminal (**Figure S20B**). Overall, these results suggest that DE proteins mainly participate in the ABC transporter type 1 transmembrane domain, glycosyl transferase, nitro reductase, and the dihydroorotate dehydrogenase domain.

### Clustering Analysis of DE Proteins

DE proteins were classified into 4 groups (Q1 to Q4) to investigate the correlation between DE protein function and differential expression fold (**Figure S21**). GO classification, KEGG enrichment analysis, protein domain enrichment analysis and clustering analysis were done in each group. According to biological process enrichment analysis, the higher differential expression fold groups (Q3 and Q4) primarily participated in glycine betaine biosynthetic or metabolic processes, DNA metabolic processes, and inorganic ion transmembrane transport; the lower differential expression fold groups (Q1 and Q2) were mainly involved in monosaccharide metabolic or biosynthetic processes, thiamine-containing compound metabolic processes, and ion homeostasis (**Figure S22A**). Cellular component analysis showed that, the Q3 and Q4 groups were in the cytosol and chromosomes and the Q1 and Q2 groups were in the cell outer membrane, external encapsulating structure, and cell envelope (**Figure S22B**). Molecular function analysis found that the Q3 and Q4 groups were mainly involved in ATPase activity, nucleotide binding or sulfur compound binding, and ion transmembrane transporter activity, whereas the Q1 and Q2 groups were mainly associated with ammonia-lyase activity, aldehyde-lyase activity and ferric iron binding (**Figure S22C**). The KEGG pathway enrichment analysis showed that the Q3 and Q4 groups were associated with inositol phosphate metabolism, RNA polymerase, quorum sensing and pyrimidine metabolism; and that the Q1 and Q2 groups participated in benzoate degradation, thiamine metabolism, degradation of aromatic compounds and nitrogen metabolism (**Figure S22D**). In the protein domain analysis, the Q3 and Q4 groups were primarily associated with peptidoglycan binding-like domain, DNA topoisomerase type IIA-like domain, the thiamine pyrophosphate enzyme, transcription regulator LuxR, C-terminal, and the ABC transporter type 1 transmembrane domain. The Q1 and Q2 groups were found to be associated with the RND efflux pump membrane fusion protein, ferritin/DPS protein domain or ferritin related domain, and the glycosyl transferase or bleomycin resistance protein (**Figure S22E**). Collectively, these results indicate that the Q3 and Q4 groups are mainly involved in biosynthetic and metabolic processes, nucleotide binding, ion transmembrane transporter activity, quorum sensing, and transcription regulator LuxR. These proteins were also found in the cytosol and chromosomes. Q1 and Q2 groups were found to be mainly involved in monosaccharide metabolic, ferric iron binding, benzoate degradation, and RND efflux pump membrane fusion protein. These proteins were also found in the cell outer membrane, external encapsulating structure, and cell envelope.

### Protein-Protein Interaction Network

All DE protein database accession or sequences were searched against the STRING database (version 10.5) for protein-protein interactions to figure out whether the DE protein had any interaction with other proteins. The hDE proteins were primarily associated with ABC transporters, quorum sensing and pyrimidine metabolism based on the KEGG database enrichment analysis. Quorum sensing associated proteins have been found to indirectly participate in *Brucella* virulence, as described previously (**Figure S13**). Furthermore, **Figure S23** shows that proteins related to ABC transporters or quorum sensing can interact with each other. For example, protein C0RMA2 can interact both with proteins C0RKU7 and A0A1Z1ZRR8. Three lDE proteins (Q8YEZ1, A0A1Z1ZJ87, and Q8YEY9) interact with each other. The detailed interaction network is shown in **Figure S23**. These results indicate that these DE proteins can interact with each other and may be associated with *Brucella* virulence.

### BtpA and vjbR Gene Expression in RAW264.7 Cells

**Figure 4:**
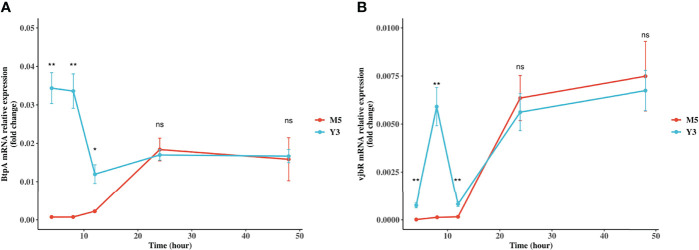
Expression of *BtpA* and *vjbR* genes in RAW264.7 cells. RAW264.7 cell lines were infected with *B. melitensis* biovar 3 or *B. melitensis* M5-90 for 4, 8, 12, 24, 48 h. *BtpA* **(A)** and *vjbR* **(B)** gene expression were analyzed using real-time quantitative RT-PCR and normalized by the expression of *16 S*.

BtpA and vjbR are two crucial virulence factors for *Brucella*. Specifically, BtpA is an exported protein produced by *Brucella* to modulate host immune response *via* interaction with TLR and vjbR is a LuxR family regulator, known to regulate virB operon (23, 24). To investigate the expression of *BtpA* and *vjbR* genes in macrophages, RAW264.7 cells were infected with *B. melitensis* biovar 3 or *B. melitensis* M5-90. The expression of BtpA and vjbR proteins in *B. melitensis* biovar 3 was higher than in *B. melitensis* M5-90 demonstrated by proteomics (**Table S3**; **Figures S24A, B**). Further validation of gene expression levels of *BtpA* and *vjbR* was performed *in vitro* at different points in time after the initial infection. These results showed that the *BtpA* gene expression in *B. melitensis* biovar 3 was 47-fold, 46-fold, and 5.5-fold higher, respectively, than in *B. melitensis* M5-90 at 4, 8, and 12 h post-infection (pi); the *vjbR* gene expression in *B. melitensis* biovar 3 was 46-fold, 45-fold, and 5-fold higher, respectively, than in *B. melitensis* M5-90 at 4, 8, and 12 h pi; from 24 h to 48 h, these two genes showed expression similar to that in *B. melitensis* biovar 3 and *B. melitensis* M5-90 (**Figures 4A, B**). BtpA and vjbR might be two reasons why the Y3 strain is more virulent than M5.

## Discussion

Brucellosis, caused by *Brucella* spp., is one of the most serious bacterial zoonotic diseases worldwide. It can infect humans, livestock, and wild animals, as well as marine animals and poses a huge threat to public health. Vaccination is still considered the most effective way to prevent the transmission of this disease. The DIVA is a key issue for vaccines currently on the market. In this study, a label-free proteomics approach was employed for the comparative evaluation of the DE proteins between two *Brucella* strains (wild strain Y3 and vaccine strain M5). Despite the high similarity of the whole genome between these two strains, many qualitative and quantitative differences were detected. A total of 147 proteins were identified as DE proteins, of which, 97 proteins were over-represented in the Y3 strain, and 50 proteins were under-expressed in the M5 strain (**Figure S2**). Unfortunately, all of the DE proteins identified in this study are not unique to the Y3 and M5 strains. Thus, these proteins are unlikely to be selected as candidate targets for definitive differentiation of vaccinated animals from wild-type infected animals. A total of 30 proteins were identified as unique to the Y3 strain and 9 proteins as unique to the M5 strain (detailed proteins are listed in **Table S2**). Hence, these unique proteins could be potential candidate markers for DIVA; however, this needs to be further validated with *in vivo* studies.

The identification and characterization of virulence factors from *Brucella* is not only crucial to understanding the biology of the pathogen, but may also provide insights into regional brucellosis epidemiology, which in turn may lead to the discovery of new targets for the development of diagnostic methods, therapeutic drugs, and vaccines (1). The published genome sequence of *B. melitensis* 16M, B. *melitensis* Rev.1, *B. melitensis* M5-90 and *B. melitensis* M28 have allowed many genomic comparisons resulting in the discovery of regions within the genome that have gene mutations or deletions resulting in many phenotypic differences among *Brucella* strains (15, 25). However, many factors involved in the virulence of a strain cannot be identified at the genomic level, and the comparison of gene expression or protein abundance between two similar species may improve our understanding.

In this study, DE proteins between Y3 and M5 *Brucella* strains were identified using a label-free proteomics approach and the differences in virulence between these two strains were defined *in vitro* and *in vivo*. It has been reported that a minor portion of DE proteins drives the diverse biological functions among *B. melitensis* strains (26). Hence, the DE proteins contribute to the distinct levels of virulence in the two strains. A total of 147 DE proteins were identified between the two samples. These DE proteins were involved in crucial processes such as metabolic processes, biosynthetic processes, and ion transmembrane transport (**Figure S8A**). The quorum sensing-dependent transcriptional regulator VjbR has been identified as an essential virulence factor in both rough-type and smooth-type *Brucella* strains (27, 28). VjbR deletion results in an attenuated phenotype in both macrophages and the mouse model. VjbR also regulates the expression of the *virB* operon and *ftcR* gene (29). In this study, the DE proteins, particularly the hDE proteins, were associated with quorum sensing (**Figures S11, S15B**). The expression of the vjbR proteins in the Y3 strain was 2-fold higher than that in the M5 strain (**Table S3**; **Figure S24B**) and the gene expression of *vjbR* in Y3 strain was also higher than in the M5 strain at the early stage of infection (**Figure 4B**). Therefore, the over-expression of the VjbR protein in the Y3 strain may contribute to its higher virulence level compared to the M5 strain. Additionally, the iron uptake system also plays a role in the virulence of *Brucella* and *pseudomonas aeruginosa*. Mutation of the iron uptake system leads to the attenuation or even an avirulent phenotype both *in vitro* and *in vivo* (30, 31). Results of KEGG database analysis in our study indicate that the *Brucella* Zur protein (encoded by gene *BMEII0175*, protein accession: Q8YDK0) is indirectly associated with iron uptake and this protein is over-represented in the Y3 strain (**Table S3**). In addition to the Irr and RirA proteins that are involved in iron transport in *Brucella* (31), Zur might be another regulatory protein involved in *Brucella* iron metabolism. It has been discovered that ABC transporters are crucial virulence factors in *Streptococcus pneumoniae* and *Escherichia coli* (32, 33). ABC transporters participate in nutrient uptake, secretion of toxins, as well as export of quorum-sensing peptides, drugs (including antibiotics) and antimicrobial peptides. Our results suggest that the ABC transporters (including mineral and organic ion transporters, phosphate and amino acid transporters and peptide and nickel transporters) are also enriched in hDE proteins (**Figure S12**). *Brucella* drug-resistance has become an urgent issue in recent years (34, 35); however, the specific mechanism(s) underlying this resistance remain unknown. Results of the current study indicate that *Brucella* might utilize quorum-sensing peptides (transported by ABC transporters) to export antibiotics from its cytoplasm. The network results also demonstrated that the ABC transporter proteins could interact with the quorum-sensing proteins (**Figure S23**). However, further research is needed to ultimately determine whether, and to what extent, quorum-sensing peptides and ABC transporters are involved in drug resistance.

Many lDE proteins were identified in the Y3 strain, meaning that these proteins are over-represented in the M5 strain. In this study, 45 proteins were identified as lDE proteins (**Figure S2**) and the majority them involved in thiamine metabolism, according to the biological process analysis and KEGG analysis (**Figures S9A, S15A**). Thiamine is also known as vitamin B1 and is required by *Listeria monocytogenes* and *Verticillium dahliae* to proliferate in host cells. The deletion of the thiamine transport gene has been found to impair the virulence and survival in hosts (36, 37). However, the function of thiamine-phosphate synthase (protein accession: Q8YEZ1) in *Brucella* remains unknown; thus, it might also be a virulence factor associated with the ability of the M5-90 strain to proliferate in hosts.

BtpA/Btp1/TcpB is a well-known virulence factor produced by *Brucella* that possesses a Toll interleukin-1 receptor (TIR) domain and can interfere with TLR signaling to modulate host immune response *via* interaction with MyD88 (a Toll-like receptor adaptor protein) (23, 38, 39). In the current study, the expression of the BtpA proteins in the Y3 strain was 8-fold higher than that in the M5 strain (**Table S3**; **Figure S24A**) and the gene expression of *BtpA* in the Y3 strain was also higher than in the M5 strain during the early stage of infection (**Figure 4A**). Hence, the BtpA protein might be an essential factor for the virulent phenotype of the Y3 strain and may be one reason for the residual virulence of the M5 strain even though the expression level is not as high as in the Y3 strain. Interestingly, in the current study, the BtpA protein could only be classified in GO secondary annotation as a cellular process, single-organism process, or binding activity (**Figure 3A**); specific information regarding KEGG database analysis or protein-protein interaction could not be found in our results.

Different virulence levels, biological functions and metabolic processes of two similar species are determined by the DE proteins, while equally abundant (EA) proteins contribute to common behaviors between two bacterial strains. A previous study compared DE and EA proteins between *B. melitensis* 16M and *B. melitensis* Rev.1 and potential biochemical routes responsible for residual virulence in the vaccine strain were revealed by analyzing the EA proteins (16). A limitation of the current study is that the EA proteins were not analyzed using GO, KEGG, and protein domain enrichment analysis. These analyses may have provided valuable information regarding the residual virulence of the M5 strain and should be completed in future work.

In summary, our data showed that the proteins unique to Y3 and M5 may be potential candidates for DIVA, though these need to be validated in animals. Furthermore, our results indicated that two well-characterized proteins, VjbR and BtpA, played a role in virulence in both the Y3 and M5 strains; however, the expressions of both proteins were lower in the M5 strain than in the Y3 strain. Additionally, results of this study indicated that the Zur protein, ABC transporters and thiamine metabolism-associated proteins may be important virulence factors involved in *Brucella* survival and pathogenesis and these three factors may also be responsible for the residual virulence of the M5 strain. However, there may be other virulence factors of importance expressed by Y3 and M5 strains. Collectively, the unique proteins identified in this study may be candidate markers for DIVA and elucidation of underlying mechanisms related to the residual virulence of the M5 vaccine strain. Further studies are required to elucidate how chronic infection with *Brucella* is established and should provide valuable information for the development of vaccines and the discovery of new therapeutic targets to control brucellosis.

## Data Availability Statement

The original contributions presented in the study are included in the article/**Supplementary Material**. Further inquiries can be directed to the corresponding authors.

## Ethics Statement

The animal study was reviewed and approved by Animal Care and Use Committee of Shihezi University.

## Author Contributions

JLS, ZW, and YZW conceived and design the experiments; CFC, YW and JHY revised the manuscript; HHL and NNY analyzed the data; ZCM and MGX contributed the reagents; HZ, YLW, TWJ, and XYD performed the experiments; HZ drafted the manuscript; YFW plotted the figure and performed the experiments. All authors contributed to the article and approved the submitted version.

## Funding

This work was supported by the National Natural Science Foundation of China (grant nos. 32002245 and U1803236).

## Conflict of Interest

The authors declare that the research was conducted in the absence of any commercial or financial relationships that could be construed as a potential or actual conflict of interest.

## Publisher’s Note

All claims expressed in this article are solely those of the authors and do not necessarily represent those of their affiliated organizations, or those of the publisher, the editors and the reviewers. Any product that may be evaluated in this article, or claim that may be made by its manufacturer, is not guaranteed or endorsed by the publisher.

## References

[1] Casadevall A Pirofski LA. Virulence Factors and Their Mechanisms of Action: The View From a Damage-Response Framework. J Water Health (2009) 7 Suppl 1:S2. doi: 10.2166/wh.2009.036 19717929

[2] Rossetti C,A Arenas-Gamboa AM Maurizio E. Caprine Brucellosis: A Historically Neglected Disease With Significant Impact on Public Health. PLoS Negl Trop Dis (2017) 11(8):e0005692. doi: 10.1371/journal.pntd.0005692 28817647 PMC5560528

[3] Berhanu G Pal M. Brucellosis: A Highly Infectious Zoonosis of Public Health and Economic Importance. J Environ Health Sci Eng (2020) 3:5–9.

[4] Liu FQ Wang DL Wang JQ Li TF Sen L. National Brucellosis Intervention Pilot County Survey on the Economic Losses. Chin J Control Endemic Dis (2008) 23:424–25. doi: 10.3969/j.issn.1001-1889.2008.06.008

[5] Liu X Yang M Song S Liu G Zhao S Liu G. Brucella Melitensis in Asian Badgers, Northwestern China. Emerging Infect Dis (2020) 26(4):804–6. doi: 10.3201/eid2604.190833 PMC7101117 32187504

[6] Wang Q Zhao S Wureli H Xie S Chen C Wei Q. Brucella Melitensis and B. Abortus in Eggs, Larvae and Engorged Females of Dermacentor Marginatus. Ticks Tick Borne Dis (2018) 9(4):1045–48. doi: 10.1016/j.ttbdis.2018.03.021 29627394

[7] Zhang H Benben W Jihai Y Zhen W Zhang J Yuanzhi W. Brucella Suis S2 Isolated From Aborted Sheep Fetuses in Northwestern China. Kafkas Univ Veteriner Fak Dergisi (2019) 25(6):869–72. doi: 10.9775/kvfd.2019.21752

[8] Zhang H Deng X Cui B Shao Z Chen C. Abortion and Various Associated Risk Factors in Dairy Cow and Sheep in Ili, China. PLoS One (2020) 15(10):e0232568. doi: 10.1371/journal.pone.0232568 33125372 PMC7598486

[9] Zhang H Shengnan S Benben W Jiang Y Wenxing W Fei G. Brucella Melitensis Isolated From Aborted Cow and Sheep Fetuses in Northwest of China. Kafkas Univ Veteriner Fak Dergisi (2018) 24(2):307–10. doi: 10.9775/kvfd.2017.18881

[10] Zhang H Xie S Wang Y Zhao X Chen C. A Case Report of Endocarditis and Spondylitis Caused by Brucella Melitensis Biovar 3. BMC Infect Dis (2021) 21(1):1–17. doi: 10.1186/s12879-021-06142-3 PMC8139066 34016047

[11] Bundle DR McGiven J. Brucellosis: Improved Diagnostics and Vaccine Insights From Synthetic Glycans. Acc Chem Res (2017) 50(12):2958–67. doi: 10.1021/acs.accounts.7b00445 PMC5738633 29219305

[12] Lalsiamthara J Lee JH. Development and Trial of Vaccines Against Brucella. J Vet Sci (2017) 18(Suppl 1):281–90. doi: 10.4142/jvs.2017.18.S1.281 PMC5583415 28859268

[13] Nielsen K. Diagnosis of Brucellosis by Serology. Vet Microbiol (2002) 90(1-4):447–59. doi: 10.1016/S0378-1135(02)00229-8 12414164

[14] Roth F Zinsstag J Orkhon D Chimed-Ochir G Otte J. Human Health Benefits From Livestock Vaccination for Brucellosis: Case Study. Bull World Health Organ (2003) 81(12):867–76. PMC2572379 14997239

[15] Eschenbrenner M Wagner MA Horn TA Kraycer JA Mujer CV Hagius S. Comparative Proteome Analysis of Brucella Melitensis Vaccine Strain Rev 1 and a Virulent Strain, 16m. J Bacteriol (2002) 184(18):4962. doi: 10.1128/JB.184.18.4962-4970.2002 12193611 PMC135307

[16] Roncada P. Comparative Proteomics of Brucella Melitensis is a Useful Toolbox for Developing Prophylactic Interventions in a One-Health Context. One Health (2021) 13(2):100253. doi: 10.1016/j.onehlt.2021.100253 33997237 PMC8100217

[17] Salmon-Divon M Kornspan D. Transcriptomic Analysis of Smooth Versus Rough Brucella Melitensis Rev.1 Vaccine Strains Reveals Insights Into Virulence Attenuation. Int J Med Microbiol (2019) 310(1):151363. doi: 10.1016/j.ijmm.2019.151363 31699441

[18] Yan X Hu S Yang Y Xu D Bu Z. Proteomics Investigation of the Time Course Responses of RAW264.7 Macrophages to Infections With the Wild-Type and Twin-Arginine Translocation Mutant Strains of Brucella Melitensis. Front Cell Infect Microbiol (2021) 11:679571. doi: 10.3389/fcimb.2021.679571 34195100 PMC8238042

[19] Zhou D Zhi F Fang J Zheng W Wang A. RNA-Seq Analysis Reveals the Role of Omp16 in Brucella-Infected RAW264.7 Cells. Front Vet Sci (2021) 8:646839. doi: 10.3389/fvets.2021.646839 33748220 PMC7970042

[20] Andrade PJ Netto MLDP Munhoz DALF Nóbrega d Hercules M Barr JR. Comparative Proteomics of Two Mycoplasma Hyopneumoniae Strains and Mycoplasma Flocculare Identified Potential Porcine Enzootic Pneumonia Determinants. Virulence (2018) 21505594:1499379–. doi: 10.1080/21505594.2018.1499379 PMC6104684 30027802

[21] Souza GD Fortuin S Aguilar D Pando RH Mcevoy C Helden PV. Using a Label-Free Proteomics Method to Identify Differentially Abundant Proteins in Closely Related Hypo- and Hypervirulent Clinical Mycobacterium Tuberculosis Beijing Isolates. Mol Cell Proteomics (2010) 9(11):2414–23. doi: 10.1074/mcp.M900422-MCP200 PMC2984234 20190197

[22] Kumar S Stecher G Tamura K. MEGA7: Molecular Evolutionary Genetics Analysis Version 7.0 for Bigger Datasets. Mol Biol Evol (2016) 33(7):1870. doi: 10.1093/molbev/msw054 27004904 PMC8210823

[23] Coronas-Serna JM Louche A Rodríguez-Escudero M Roussin M Imbert PR Rodríguez-Escudero I. The TIR-Domain Containing Effectors BtpA and BtpB From Brucella Abortus Impact NAD Metabolism. PLoS Pathog (2020) 16(4):e1007979. doi: 10.1371/journal.ppat.1007979 32298382 PMC7188309

[24] Jong MFD Sun YH Hartigh ABD Dijl JMV Tsolis RM. Identification of VceA and VceC, Two Members of the VjbR Regulon That are Translocated Into Macrophages by the Brucella Type IV Secretion System. Mol Microbiol (2010) 70(6):1378–96. doi: 10.1111/j.1365-2958.2008.06487.x PMC2993879 19019140

[25] Wang F Qiao Z Hu S Liu W Zheng H Liu S. Comparison of Genomes of Brucella Melitensis M28 and the B. Melitensis M5-90 Derivative Vaccine Strain Highlights the Translation Elongation Factor Tu Gene Tuf2 as an Attenuation-Related Gene. Infect Immun (2013) 81(8):2812–8. doi: 10.1128/IAI.00224-13 PMC3719578 23716607

[26] Rahman N Shah M Muhammad I Khan H Imran M. Genome-Wide Core Proteome Analysis of Brucella Melitensis Strains for Potential Drug Target Prediction. Mini Rev Med Chem (2021) 21(18):2778–87. doi: 10.2174/1389557520666200707133347 32634082

[27] Delrue RM Deschamps C Léonard S Nijskens C Danese I Schaus JM. A Quorum-Sensing Regulator Controls Expression of Both the Type IV Secretion System and the Flagellar Apparatus of Brucella Melitensis. Cell Microbiol (2005) 7(8):1151–61. doi: 10.1111/j.1462-5822.2005.00543.x 16008582

[28] Clausse M Diaz AG Ghersi G Zylberman V Cassataro J Giambartolomei GH. The vaccine candidate BLSOmp31 protects mice against Brucella canis infection - ScienceDirect. Vaccine (2013) 31(51):6129–35. doi: 10.1016/j.vaccine.2013.07.041 23906889

[29] Liu Y Sun J Peng X Dong H Qin Y Shen Q. Deletion of the LuxR-Type Regulator VjbR in Brucella Canis Affects Expression of Type IV Secretion System and Bacterial Virulence, and the Mutant Strain Confers Protection Against Brucella Canis Challenge in Mice. Microb Pathog (2020) 139:103865. doi: 10.1016/j.micpath.2019.103865 31715318

[30] Minandri F Imperi F Frangipani E Bonchi C Visaggio D Facchini M. Role of Iron Uptake Systems in Pseudomonas Aeruginosa Virulence and Airway Infection. Infect Immun (2016) 84(8):2324–35. doi: 10.1128/IAI.00098-16 PMC4962624 27271740

[31] Zhang H Wang B Wu W Deng X Shao Z Yi J. Insights Into Irr and rirA Gene Regulation on the Virulence of Brucella Melitensis M5-90. Can J Microbiol (2020) 66(5):351–358. doi: 10.1139/cjm-2019-0393 32040345

[32] Durmort C Brown JS. Streptococcus Pneumoniae Lipoproteins and ABC Transporters. Streptococcus Pneumoniae: Elsevier (2015) 181–206. doi: 10.1016/B978-0-12-410530-0.00010-7

[33] Kaul G Pattan G. MsbA ATP-Binding Cassette (ABC) Transporter of E. Coli: Structure and Possible Flippase Mechanism. Indian J Biochem Biophys (2011) 48(1):7–13. 21469596

[34] Johansen TB Scheffer L Jensen VK Bohlin J Feruglio SL. Whole-Genome Sequencing and Antimicrobial Resistance in Brucella Melitensis From a Norwegian Perspective. Sci Rep (2018) 8(1):1–9. doi: 10.1038/s41598-018-26906-3 29867163 PMC5986768

[35] Shevtsov A Syzdykov M Kuznetsov A Shustov A Shevtsova E Berdimuratova K. Antimicrobial Susceptibility of Brucella Melitensis in Kazakhstan. Antimicrob Resist Infect Control (2017) 6(1):1–5. doi: 10.1186/s13756-017-0293-x PMC5745643 29299304

[36] Qi X Su X Guo H Qi J Cheng H. VdThit, a Thiamine Transport Protein, is Required for Pathogenicity of the Vascular Pathogen Verticillium Dahliae. Mol Plant-Microbe Interact (2016) 29(7):545–59. doi: 10.1094/MPMI-03-16-0057-R 27089469

[37] Schauer K Stolz Scherer S Fuchs TM. Both Thiamine Uptake and Biosynthesis of Thiamine Precursors are Required for Intracellular Replication of Listeria Monocytogenes. J Bacteriol (2009) 191(7):2218–27. doi: 10.1128/JB.01636-08 PMC2655527 19181806

[38] Kaplan-Türk?z B Koelblen T Felix C MP C O’Callaghan D AC V. Structure of the Toll/interleukin 1 Receptor (TIR) Domain of the Immunosuppressive Brucella Effector BtpA/Btp1/TcpB. FEBS Lett (2013) 587(21):3412–6. doi: 10.1016/j.febslet.2013.09.007 24076024

[39] Snyder GA Deredge D Waldhuber A Fresquez T Wilkins DZ Smith PT. Crystal Structures of the Toll/Interleukin-1 Receptor (TIR) Domains From the Brucella Protein TcpB and Host Adaptor TIRAP Reveal Mechanisms of Molecular Mimicry. J Biol Chem (2014) 289(2):669–79. doi: 10.1074/jbc.M113.523407 PMC3887195 24275656

