# Supplementary material for: Using a Relative Quantitative Proteomic Method to Identify Differentially Abundant Proteins in Brucella melitensis Biovar 3 and Brucella melitensis M5-90
Source: Front Immunol. 2022 Jul 19;13:929040. doi: 10.3389/fimmu.2022.929040 (PMC9343586; doi:10.3389/fimmu.2022.929040)
Supplement: Supplementary file 1 [file DataSheet_1.zip › Supplementary Figure Legends.docx]

**Supplementary Figure 1.** Data analysis from LC-MS/MS results. (A) The bar plot shows the number of identified total spectra, peptides and proteins; (B) Venn diagram displaying the overlap of common and unique proteins to *B. melitensis* biovar 3 and *B. melitensis* M5-90; the overlap section represents the 2,094 common proteins between these two strains; the blue section represents 9 proteins unique to *B. melitensis* M5-90, the orange section represents 30 unique proteins to *B. melitensis* biovar 3.

**Supplementary Figure 2.** Identification of DE proteins of *B. melitensis* biovar 3 and *B. melitensis* M5-90. The bar plot shows the 97 hDE proteins (red) and 50 lDE proteins (blue).

**Supplementary Figure 3.** Repeatability examination of *B. melitensis* biovar 3 and *B. melitensis* M5-90 bacterial samples. (A) Dataset distribution over a PCA plot on a bacterial strain basis; identified proteins are scattered onto a PCA plot on a bacterial strain basis. (B) A box plot was used to compare the intensity distribution of these two bacterial samples and the RSD value indicates the repeatability of the data. Each group has three replicates.

**Supplementary Figure 4.** Classification of GO secondary annotation of lDE and hDE proteins. The biological function of lDE proteins (A) and hDE proteins (B) were characterized by biological process, cellular component, and molecular function.

**Supplementary Figure 5.** Subcellular location of lDE proteins and hDE proteins. Pie chart shows the proportion of subcellular location of lDE proteins (A) and hDE proteins (B).

**Supplementary Figure 6.** COG/KOG function categorization of DE proteins. The bar plot shows the specific function of DE proteins of *B. melitensis* biovar 3 and *B. melitensis* M5-90 based on the COG or KOG database analysis.

**Supplementary Figure 7.** COG/KOG function categorization of lDE proteins and hDE proteins. Bar plot shows the specific function of lDE proteins (A) and hDE proteins (B) based on the COG or KOG database analysis.

**Supplementary Figure 8.** GO functional categorization of the DE proteins. The bubble plot shows the biological process (A), cellular component (B), and molecular function (C) of the DE proteins of *B. melitensis* biovar 3 and *B. melitensis* M5-90 according to the GO functional categorization.

**Supplementary Figure 9.** GO functional categorization of the lDE proteins and hDE proteins. Bubble plot shows the biological process of lDE proteins (A) and hDE proteins (B); cellular component of lDE proteins (C) and hDE proteins (D); and the molecular function of lDE proteins (E) and hDE proteins (F).

**Supplementary Figure 10.** Directed acyclic graph of DE proteins based on the GO enrichment analysis. Directed acyclic graph of DE proteins (A); Directed acyclic graph of lDE proteins (B) and hDE proteins (C). The red circle represents the GO enrichment analysis with the extremely significant DE proteins (*P* < 0.01); yellow circle represents the GO enrichment analysis with the significant DE proteins (*P* < 0.05); and blue circle represents the GO enrichment analysis with the non-significant DE proteins (*P* > 0.05). The lines with arrows indicate the hierarchy of GO classification. The size of the circle represents the degree of enrichment.

**Supplementary Figure 11.** KEGG pathway enrichment analysis of DE proteins. The bubble plot shows the specific functions of DE proteins according to the KEGG pathway enrichment analysis.

**Supplementary Figure 12.** Detailed KEGG pathway enrichment analysis of hDE proteins in ABC transporters.

**Supplementary Figure 13.** Detailed KEGG pathway enrichment analysis of hDE proteins in quorum sensing.

**Supplementary Figure 14.** Detailed KEGG pathway enrichment analysis of hDE proteins in pyrimidine metabolism.

**Supplementary Figure 15.** KEGG pathway enrichment analysis of lDE proteins and hDE proteins. Bubble plot shows the KEGG pathway enrichment analysis of lDE proteins (A) and hDE proteins (B).

**Supplementary Figure 16.** Detailed KEGG pathway enrichment analysis of lDE proteins in thiamine metabolism.

**Supplementary Figure 17.** Detailed KEGG pathway enrichment analysis of lDE proteins in degradation of aromatic compounds.

**Supplementary Figure 18.** Detailed KEGG pathway enrichment analysis of lDE proteins in benzoate degradation.

**Supplementary Figure 19.** Protein domain categorization of DE proteins. The bubble plot shows the specific domain enrichment analysis of DE proteins of *B. melitensis* biovar 3 and *B. melitensis* M5-90.

**Supplementary Figure 20.** Protein domain categorization of lDE and hDE proteins. Bubble plot shows the domain enrichment analysis of lDE proteins (A) and hDE proteins (B).

**Supplementary Figure 21.** The number of proteins in classified groups. DE proteins were classified into 4 groups (Q1 to Q4) according to the differential expression fold.

**Supplementary Figure 22.** Clustering analysis of DE proteins based on the GO categorization, KEGG pathway enrichment and protein domain enrichment analysis. Heatmap shows the specific biological process (A), cellular component (B), molecular function (C), KEGG pathway enrichment analysis (D), and protein domain analysis (E) of the four groups (Q1 to Q4). Red represents a strong degree of enrichment; blue represents weak enrichment.

**Supplementary Figure 23.** Protein-protein interaction network graph. Protein accession was obtained from screening different groups (Q1 to Q4) and searching against the STRING (v.10.5) database to obtain differential protein interactions according to the confidence > 0.7 (high confidence). R (v.4.0.5) package “networkD3” was used to construct the protein-protein network graph. Red circles represent over-expressed proteins, blue circles represent down-regulated proteins. The circle size represents the number of proteins interacting with DE proteins. The top 50 interactions were selected to construct the interaction network.

**Supplementary Figure 24.** Expression of BtpA and VjbR protein in *Brucella melitensis* biovar 3 and *Brucella melitensis* M5-90. The label-free quantification (LFQ) intensity of protein in each sample was obtained by LFQ calculation and the value of relative quantification was determined based on the different LFQ intensities in each sample. Each group has three replicates.
