# Supplementary material for: Using a Relative Quantitative Proteomic Method to Identify Differentially Abundant Proteins in Brucella melitensis Biovar 3 and Brucella melitensis M5-90
Source: Front Immunol. 2022 Jul 19;13:929040. doi: 10.3389/fimmu.2022.929040 (PMC9343586; doi:10.3389/fimmu.2022.929040)
Supplement: Supplementary file 1 [file DataSheet_1.zip › Supplementary Table 1.docx]

**Supplementary Table 1**. Quantification of extracted sample proteins

| No. of samples | Protein concentration(μg/μL) | Volume of sample (μL) | Amount of protein(μg) |
| --- | --- | --- | --- |
| M5-1 | 1.1 | 1200 | 1323.5 |
| M5-2 | 1.27 | 1200 | 1519.1 |
| M5-3 | 1.17 | 1200 | 1405.2 |
| Y3-1 | 0.76 | 1200 | 907.7 |
| Y3-2 | 1.17 | 1200 | 1407.7 |
| Y3-3 | 0.8 | 1200 | 964.6 |
