# Supplementary figures and images for: Using a Relative Quantitative Proteomic Method to Identify Differentially Abundant Proteins in Brucella melitensis Biovar 3 and Brucella melitensis M5-90
Source: Front Immunol. 2022 Jul 19;13:929040. doi: 10.3389/fimmu.2022.929040 (PMC9343586; doi:10.3389/fimmu.2022.929040)

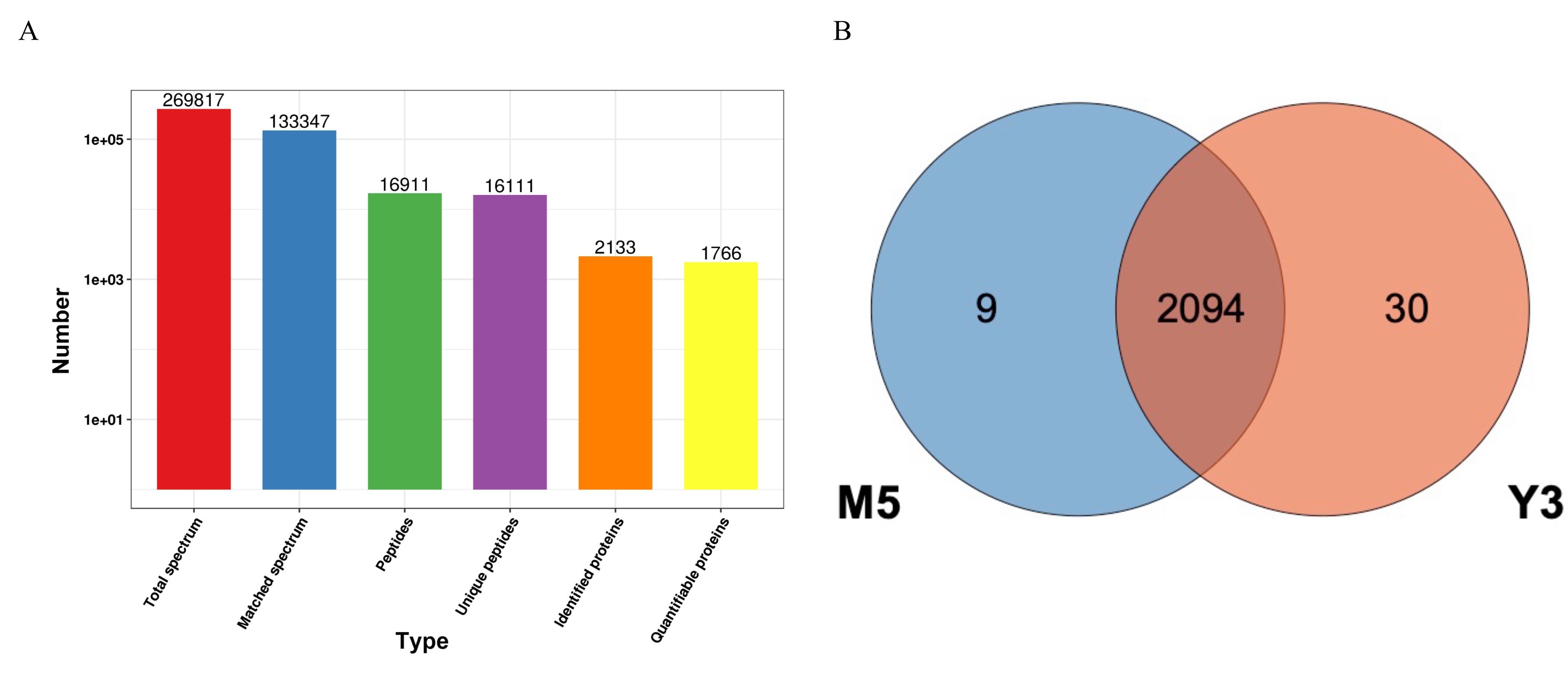

Supplement: Supplementary file 1 [file DataSheet_1.zip › Supplementary Figure 1.jpg]

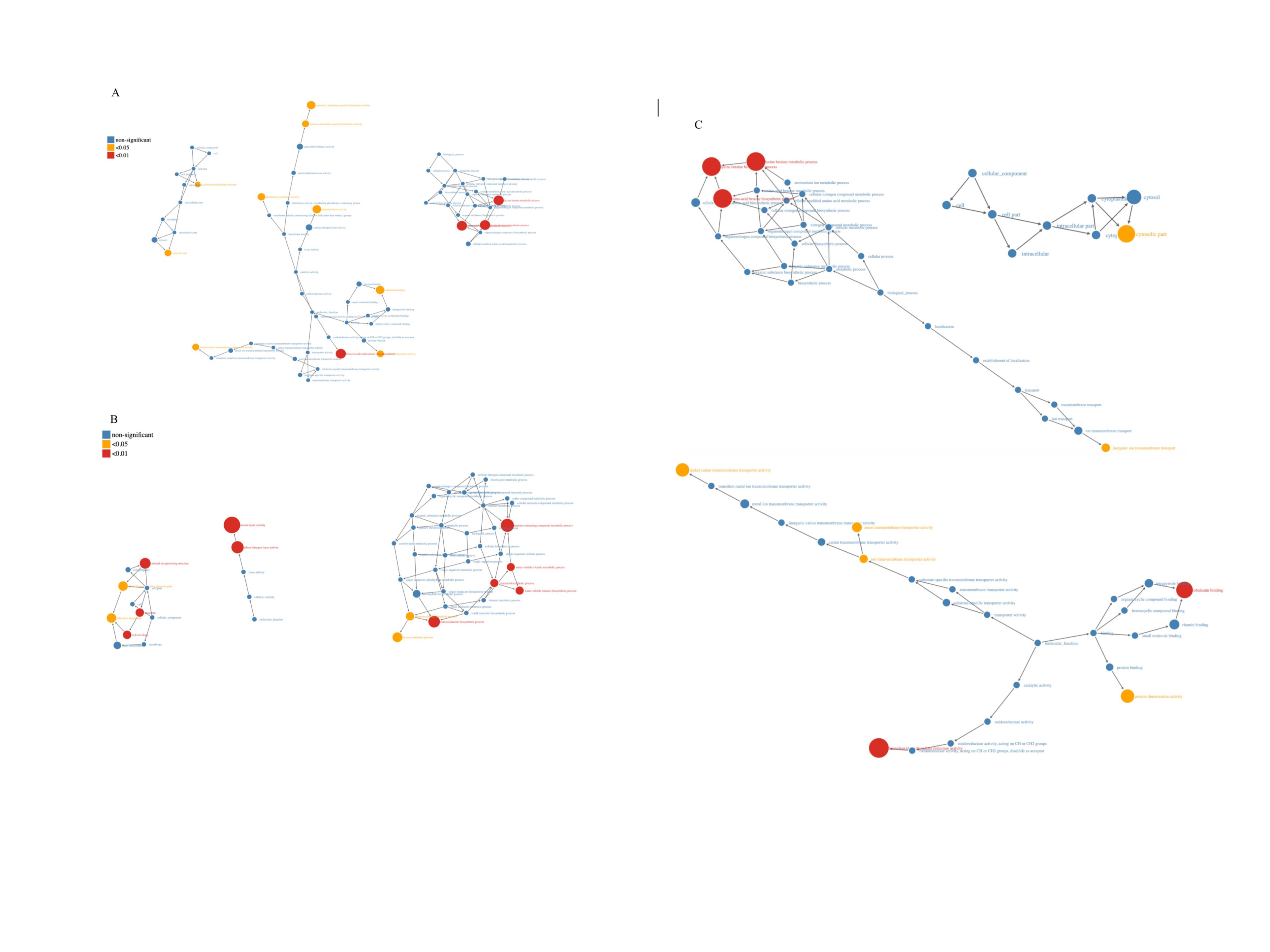

Supplement: Supplementary file 1 [file DataSheet_1.zip › Supplementary Figure 10.jpg]

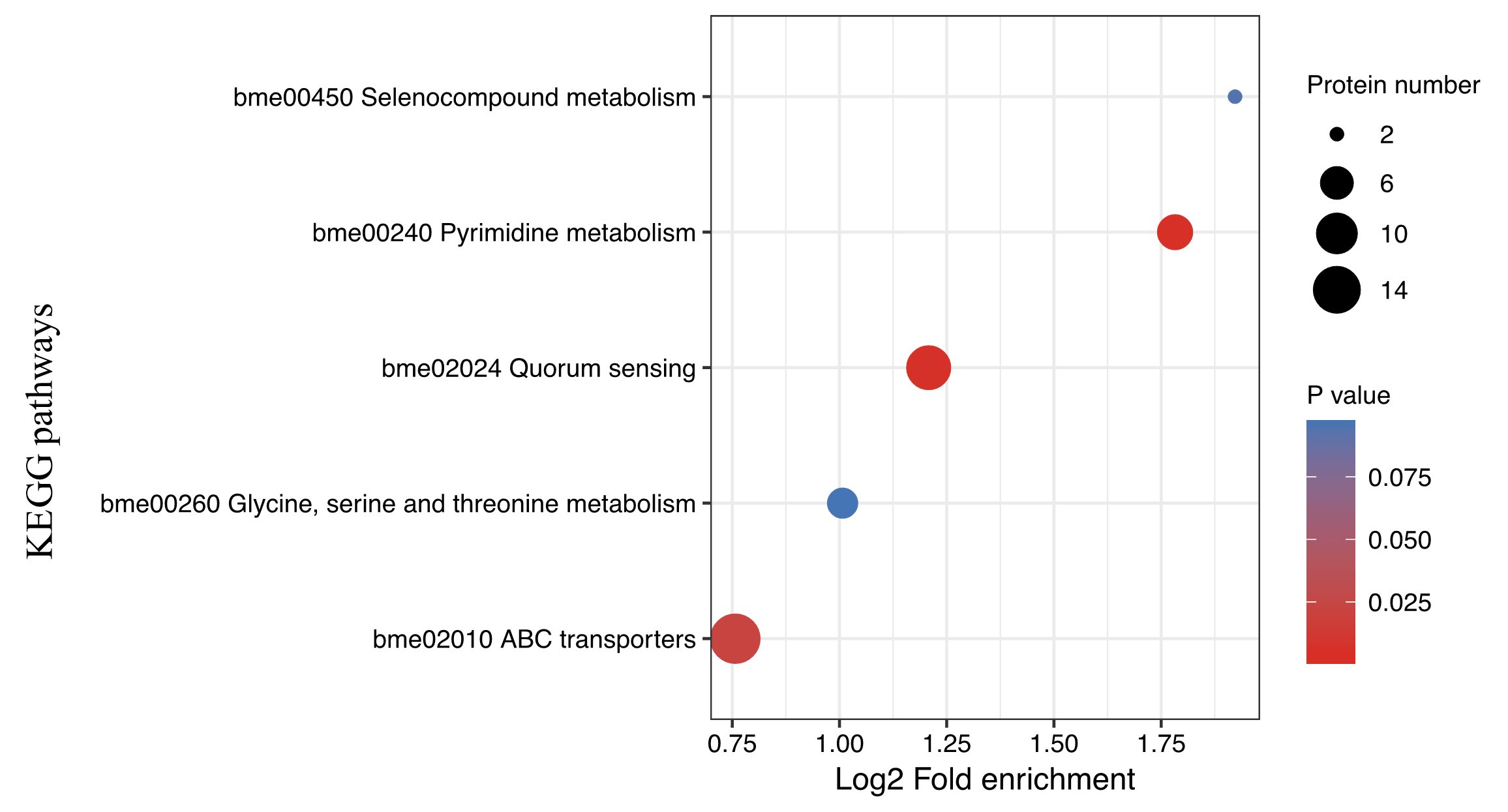

Supplement: Supplementary file 1 [file DataSheet_1.zip › Supplementary Figure 11.jpg]

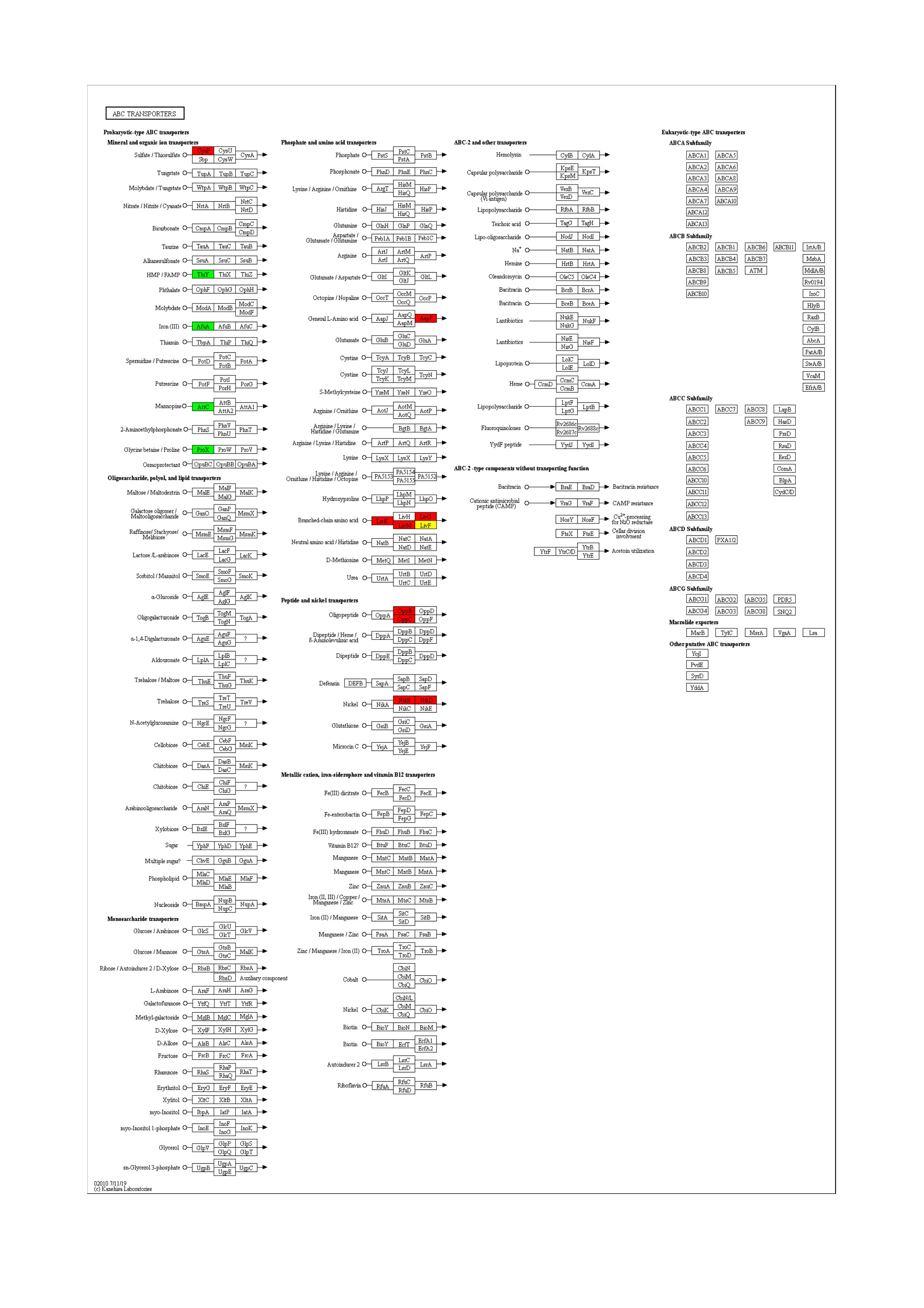

Supplement: Supplementary file 1 [file DataSheet_1.zip › Supplementary Figure 12.jpg]

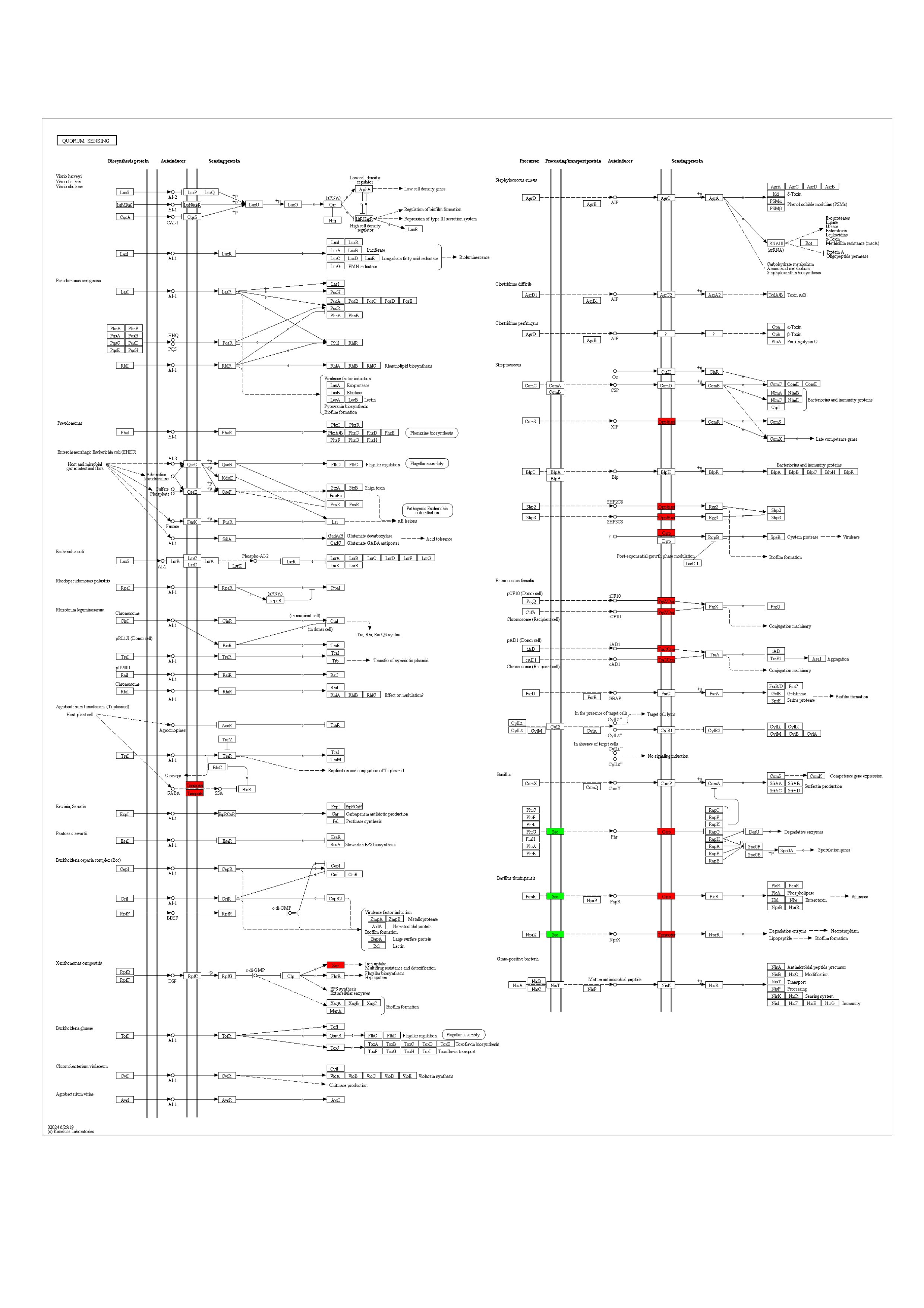

Supplement: Supplementary file 1 [file DataSheet_1.zip › Supplementary Figure 13.jpg]

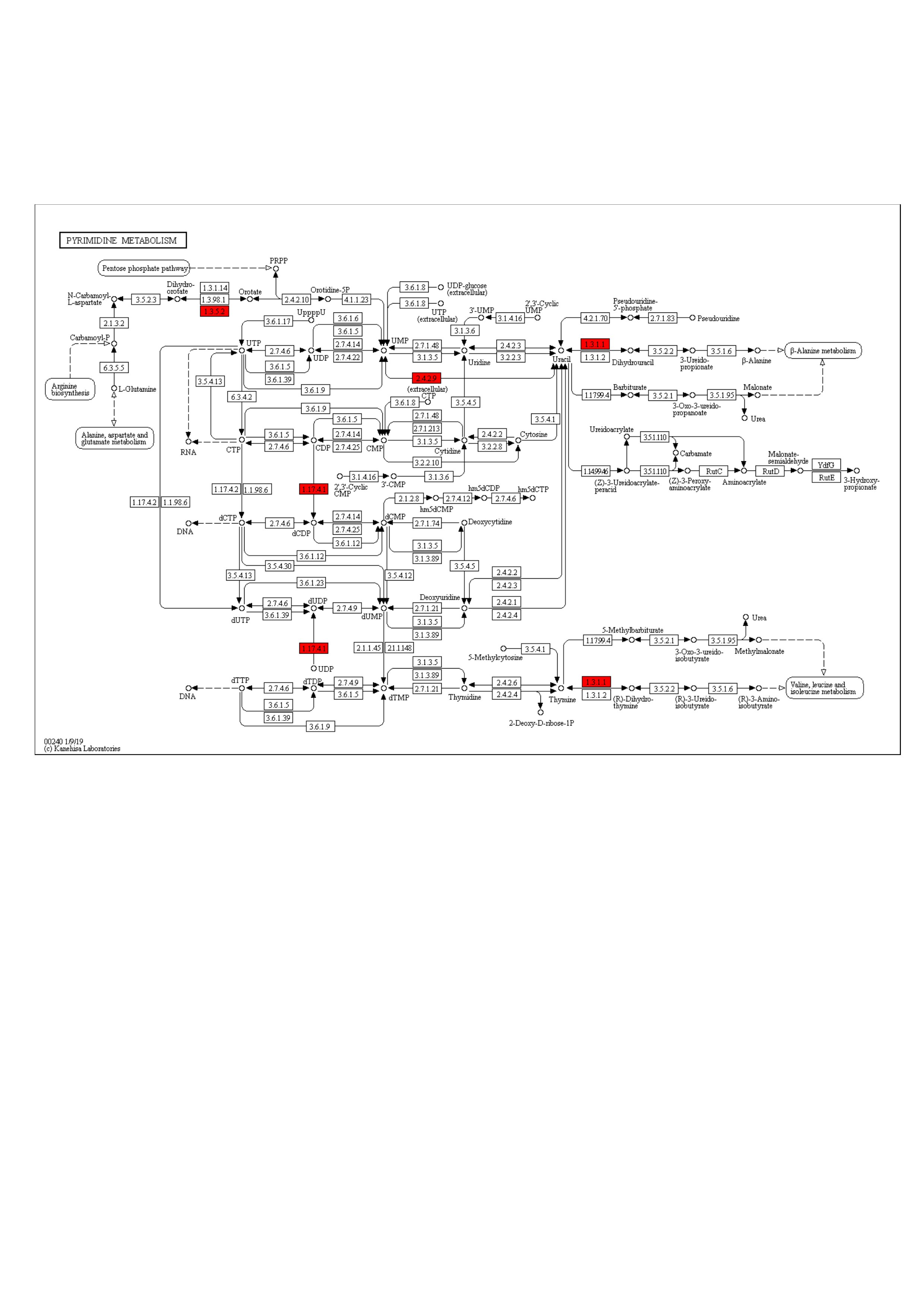

Supplement: Supplementary file 1 [file DataSheet_1.zip › Supplementary Figure 14.jpg]

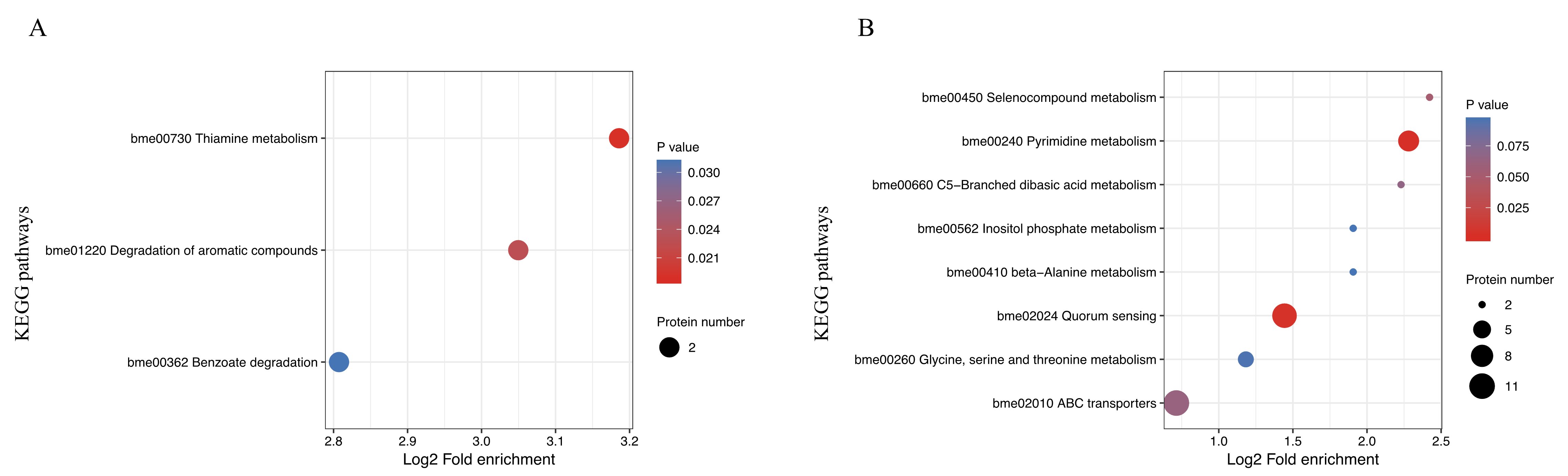

Supplement: Supplementary file 1 [file DataSheet_1.zip › Supplementary Figure 15.jpg]

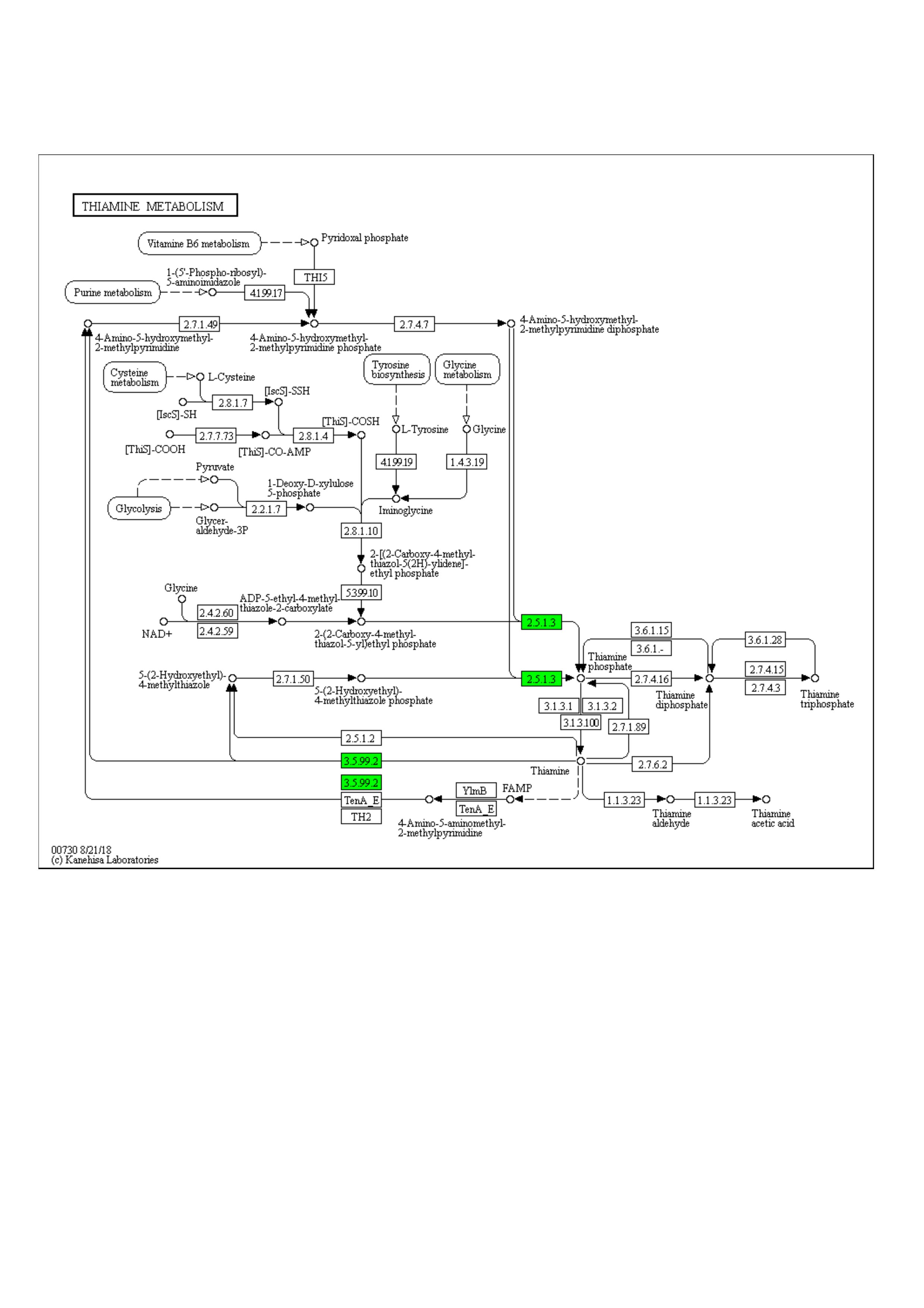

Supplement: Supplementary file 1 [file DataSheet_1.zip › Supplementary Figure 16.jpg]

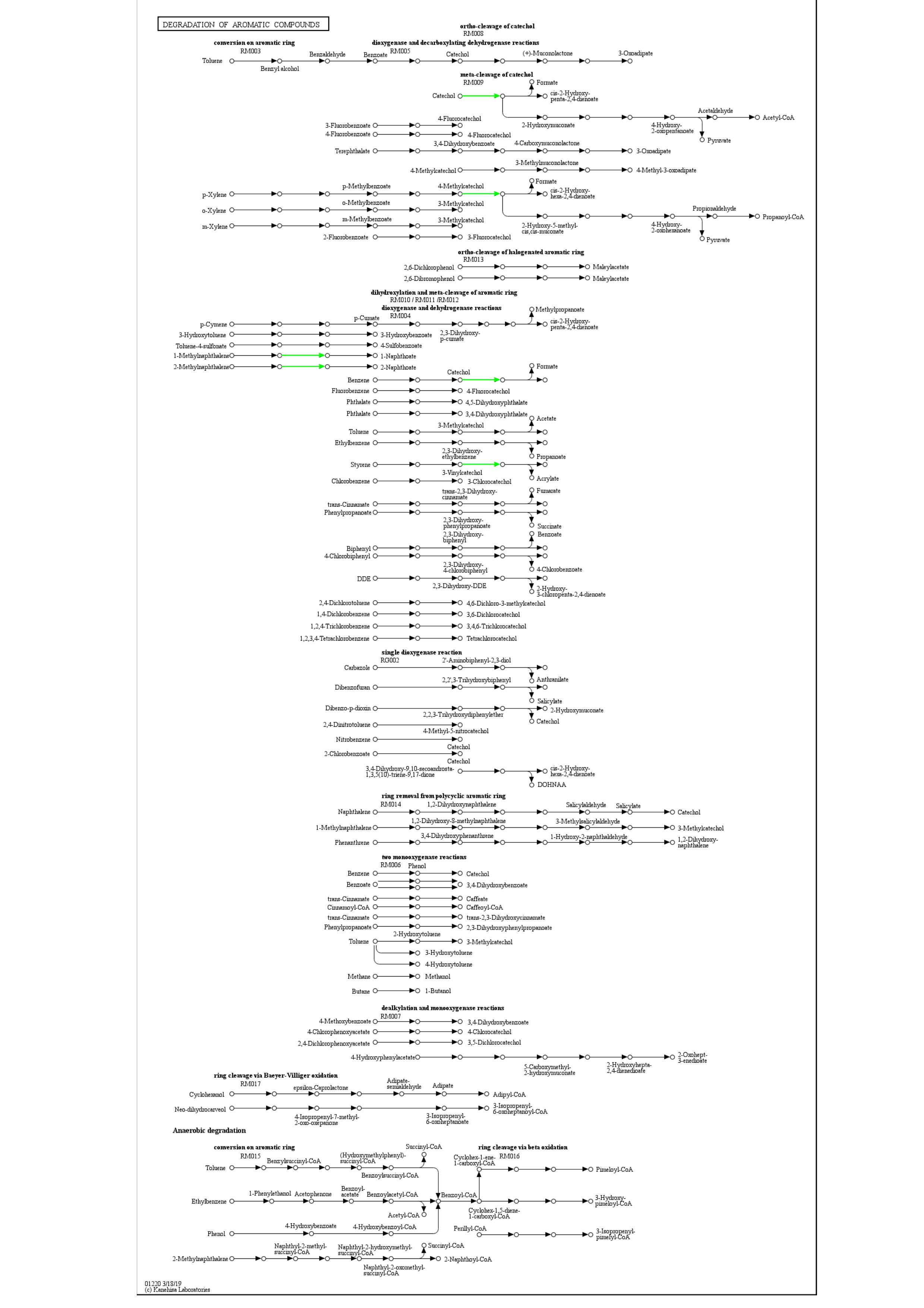

Supplement: Supplementary file 1 [file DataSheet_1.zip › Supplementary Figure 17.jpg]

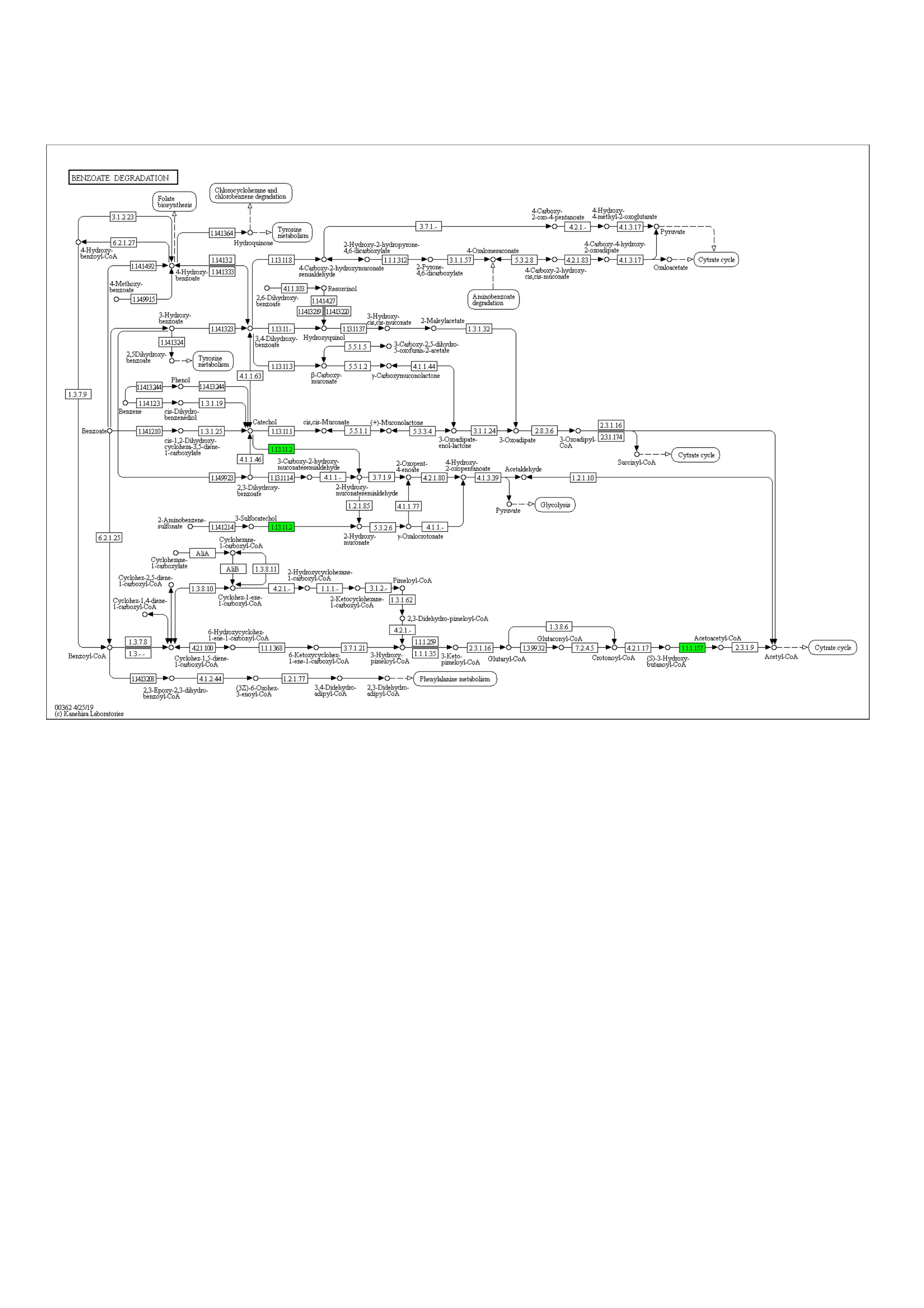

Supplement: Supplementary file 1 [file DataSheet_1.zip › Supplementary Figure 18.jpg]

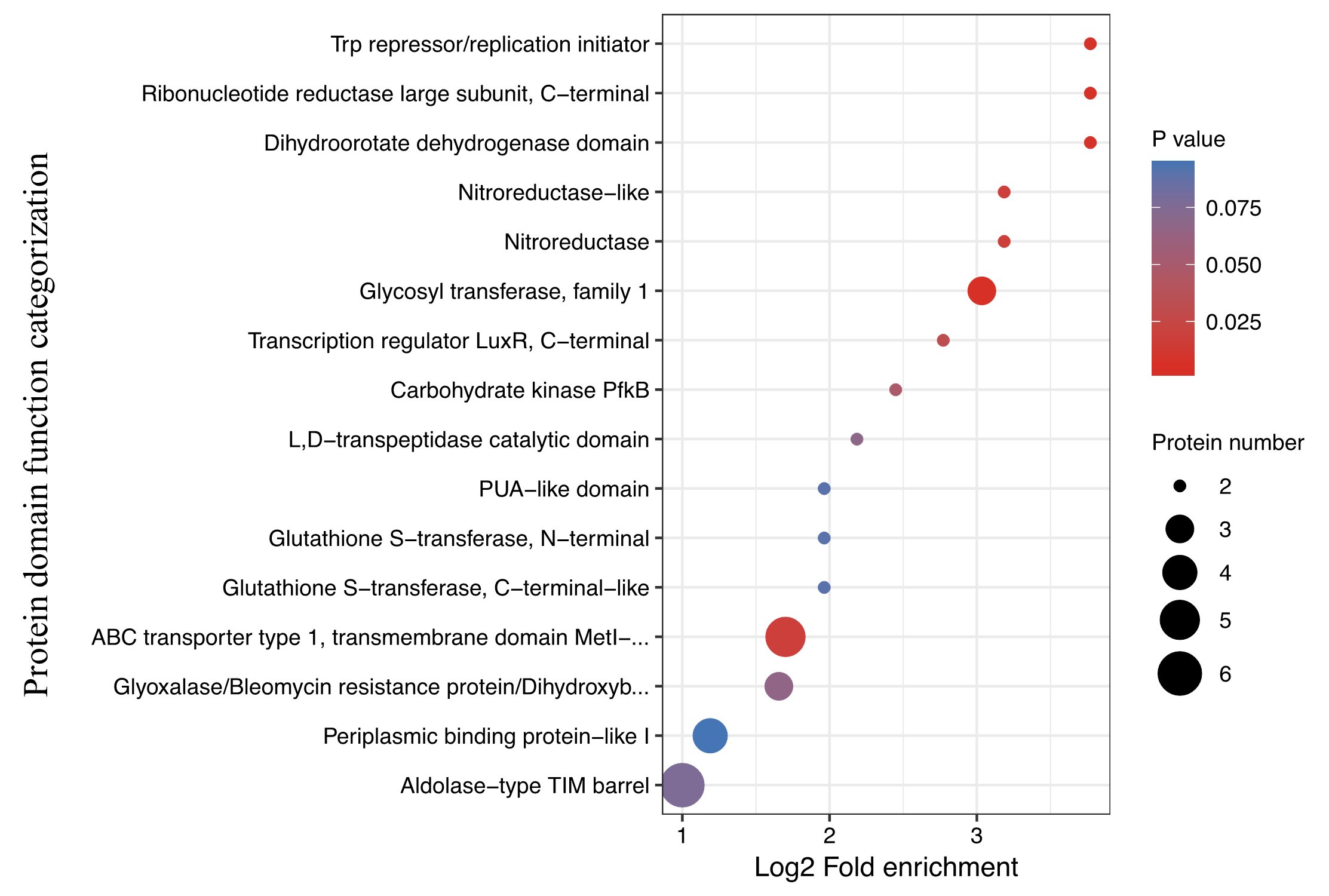

Supplement: Supplementary file 1 [file DataSheet_1.zip › Supplementary Figure 19.jpg]

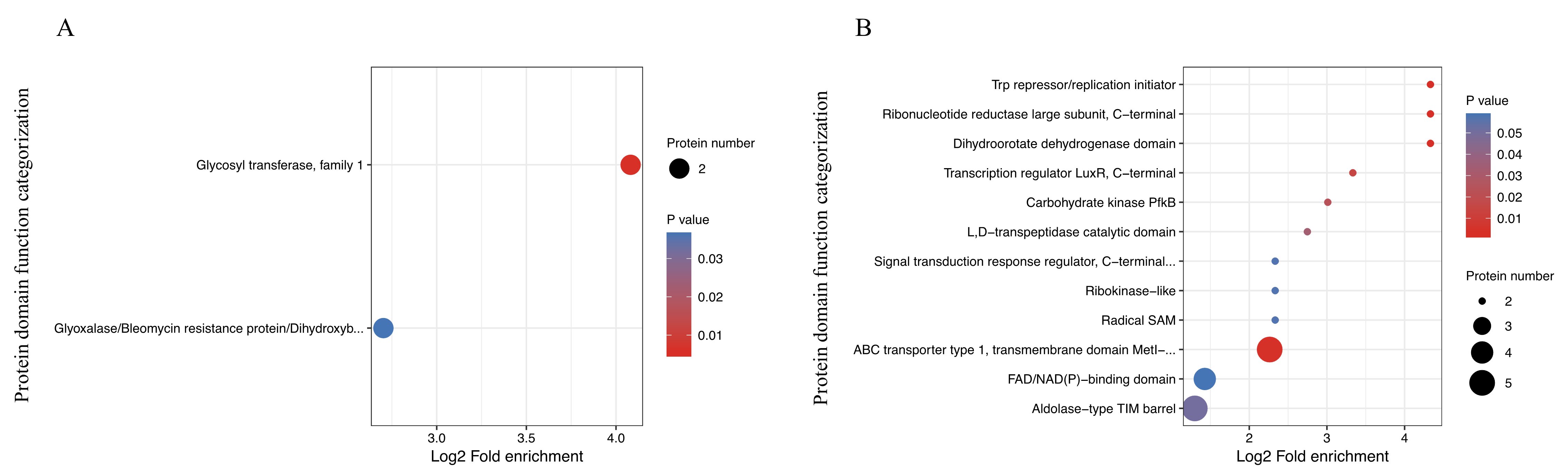

Supplement: Supplementary file 1 [file DataSheet_1.zip › Supplementary Figure 20.jpg]

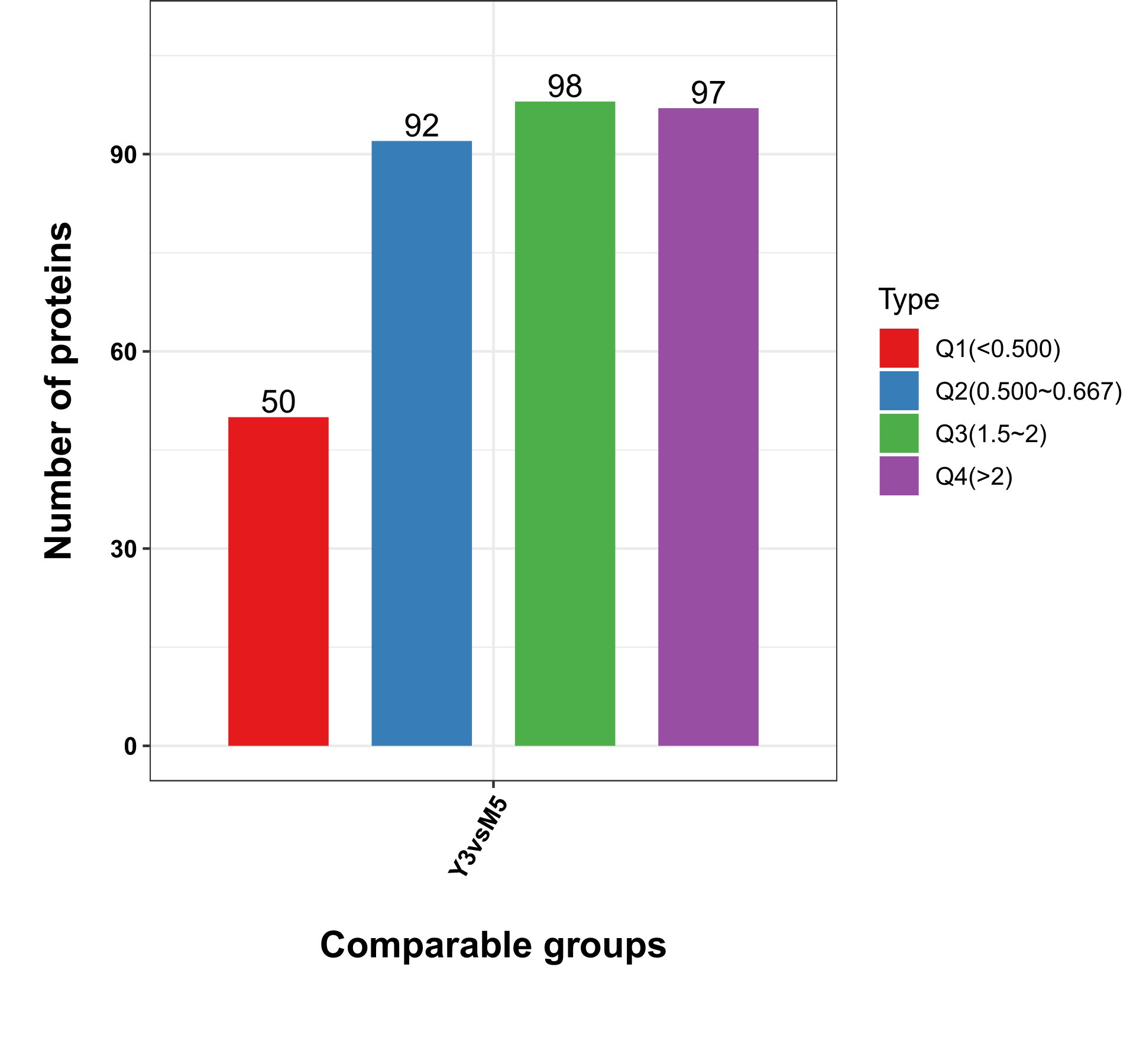

Supplement: Supplementary file 1 [file DataSheet_1.zip › Supplementary Figure 21.jpg]

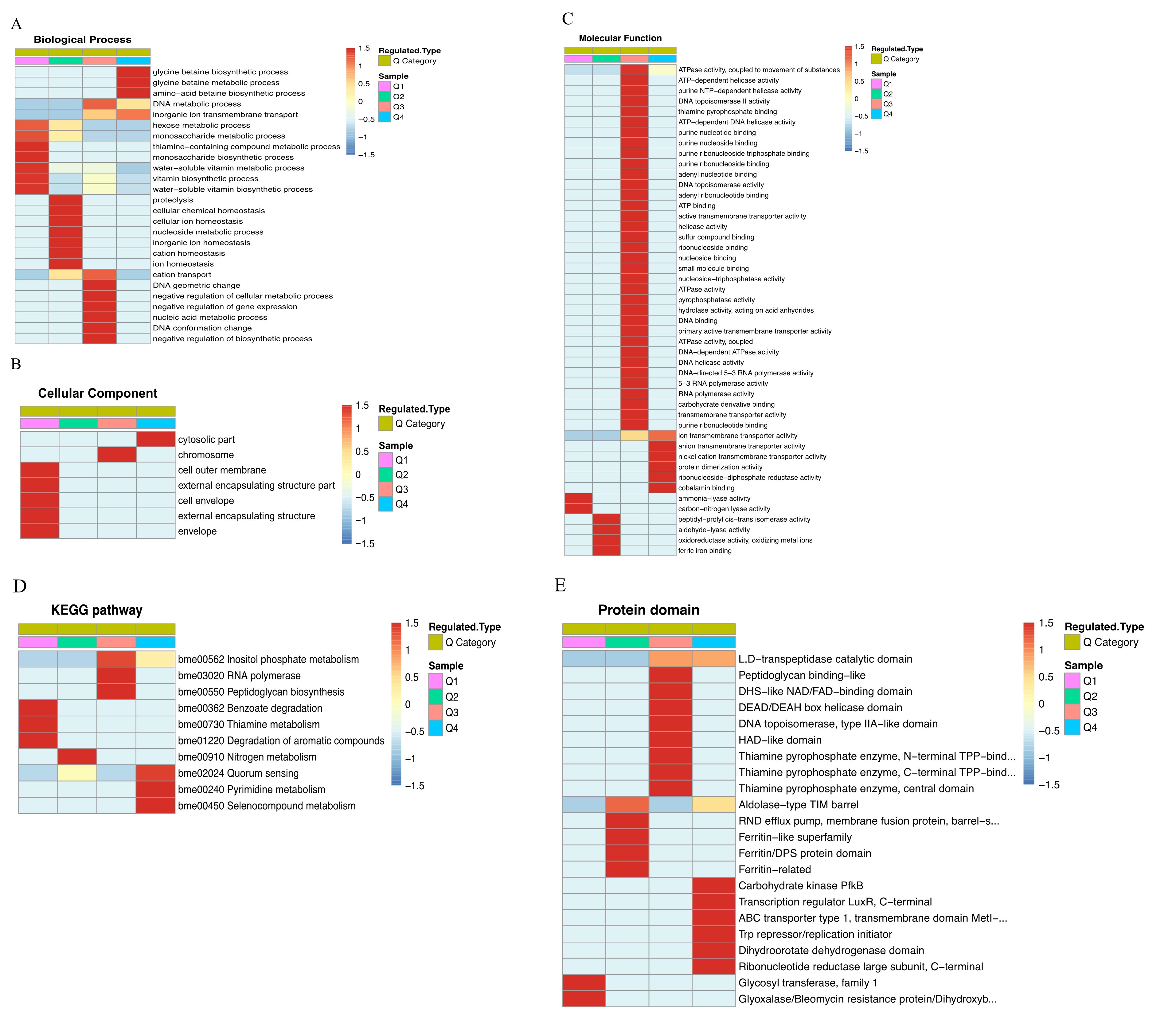

Supplement: Supplementary file 1 [file DataSheet_1.zip › Supplementary Figure 22.jpg]

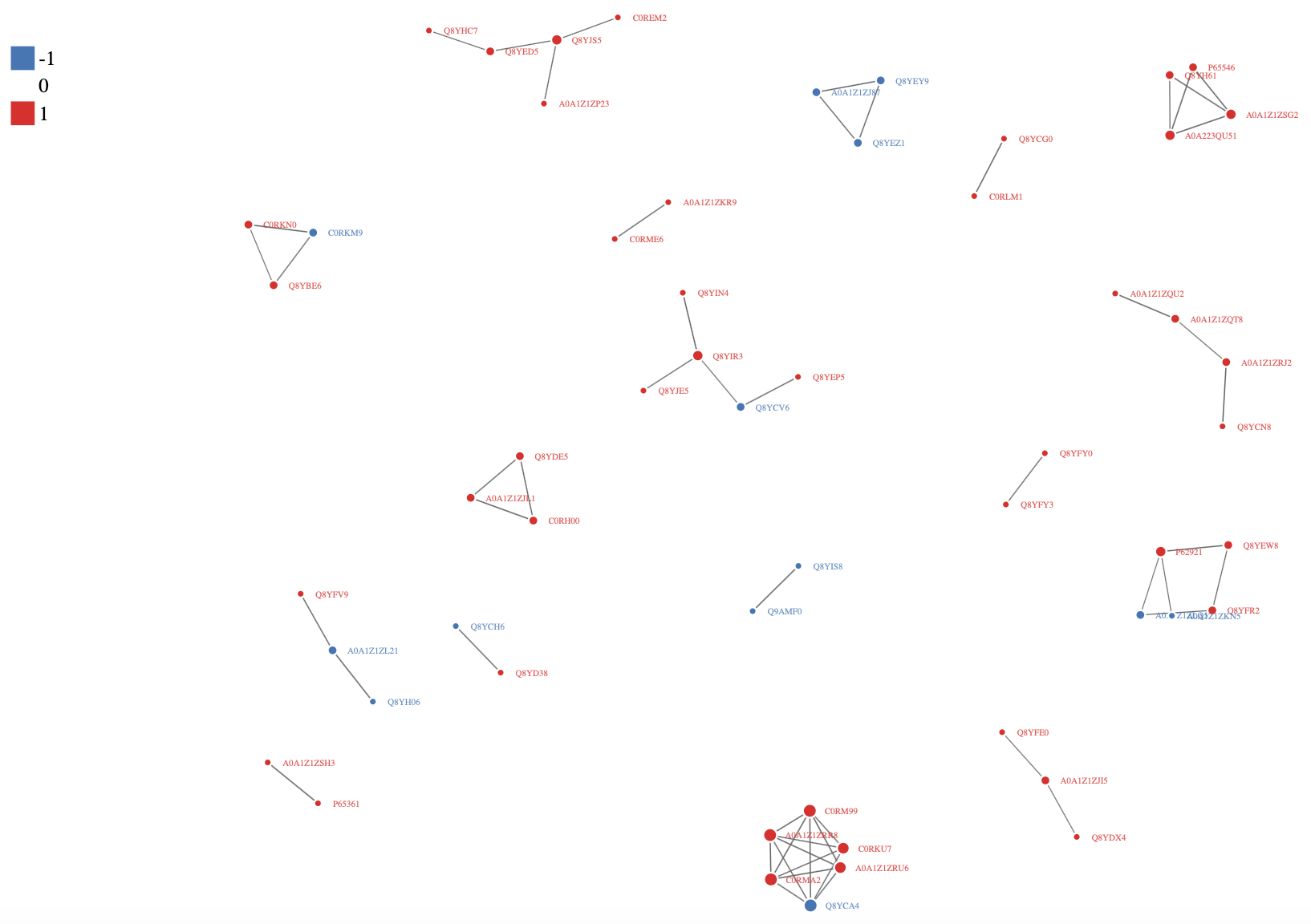

Supplement: Supplementary file 1 [file DataSheet_1.zip › Supplementary Figure 23.jpg]

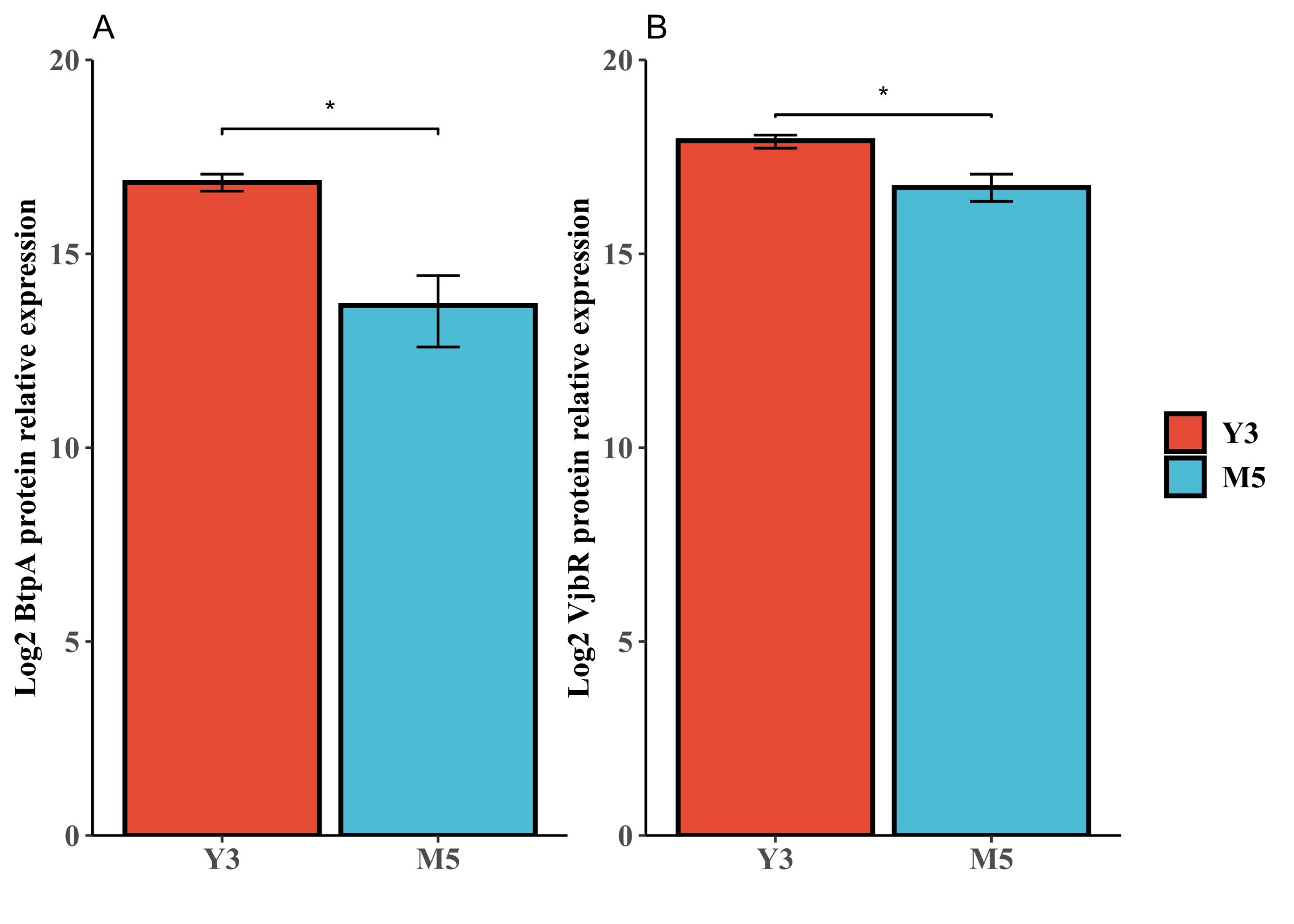

Supplement: Supplementary file 1 [file DataSheet_1.zip › Supplementary Figure 24.jpg]

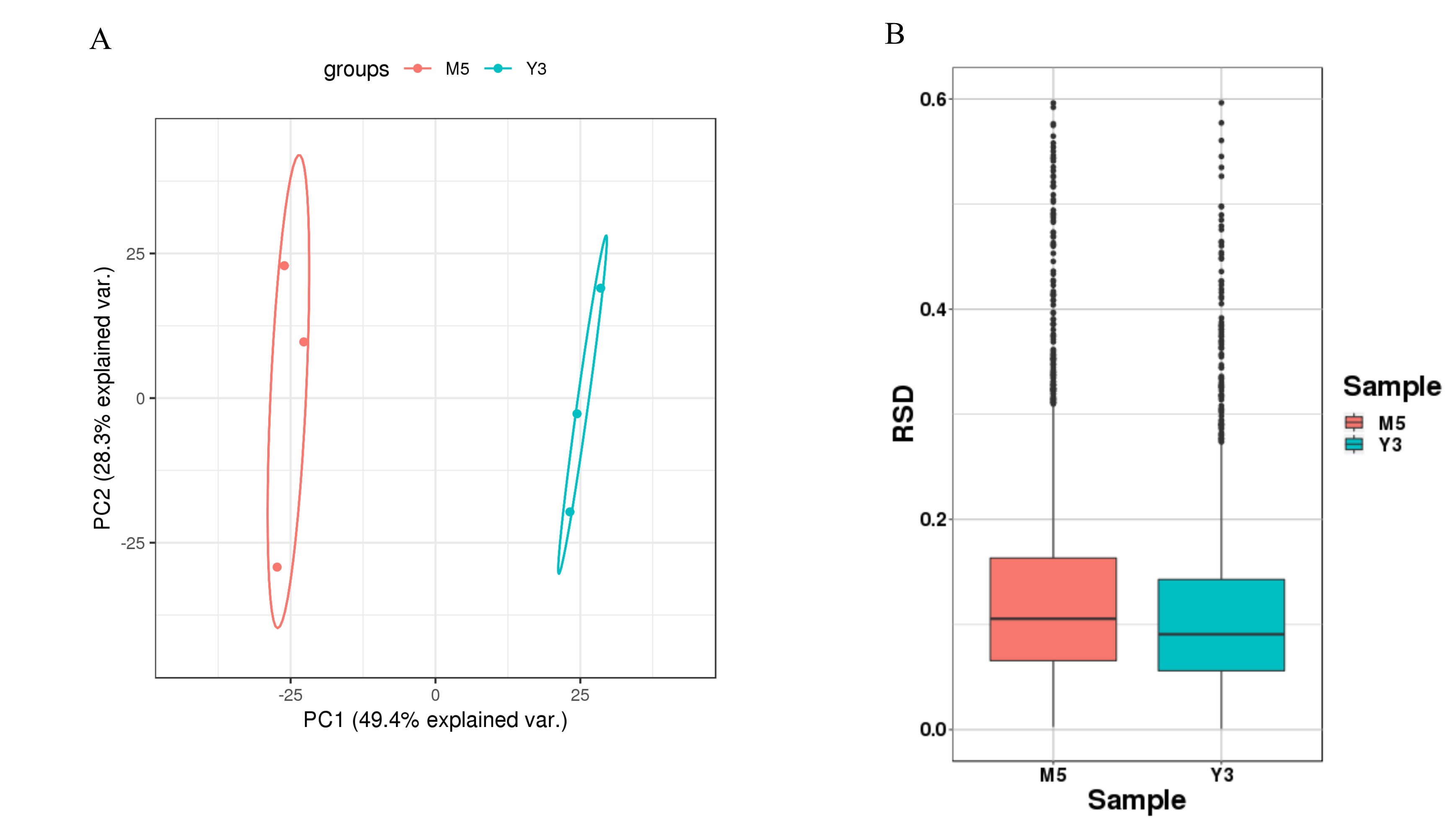

Supplement: Supplementary file 1 [file DataSheet_1.zip › Supplementary Figure 3.jpg]

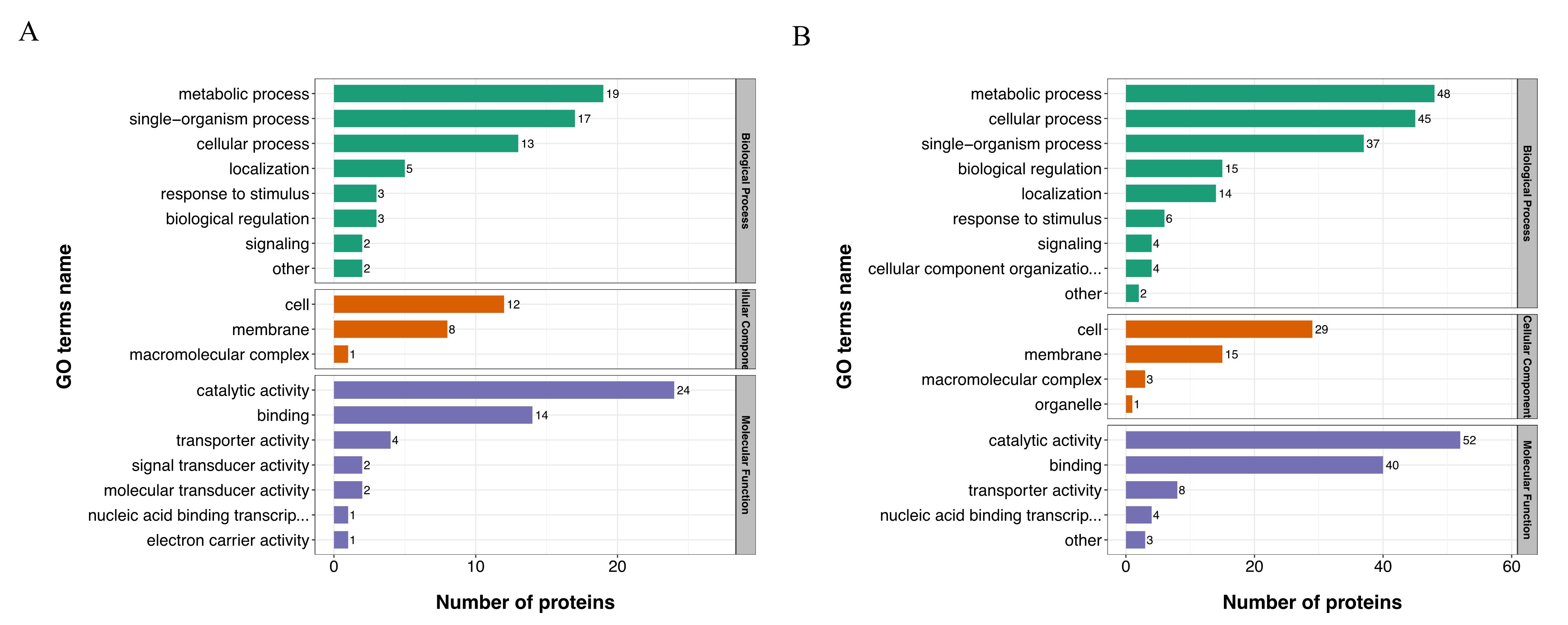

Supplement: Supplementary file 1 [file DataSheet_1.zip › Supplementary Figure 4.jpg]

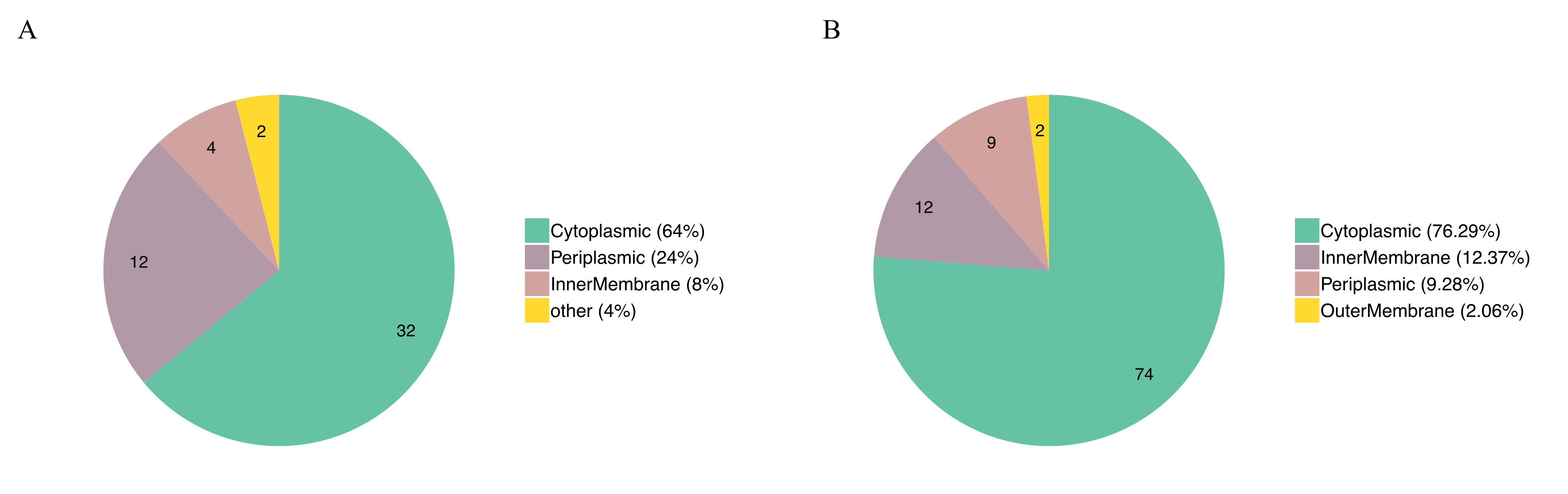

Supplement: Supplementary file 1 [file DataSheet_1.zip › Supplementary Figure 5.jpg]

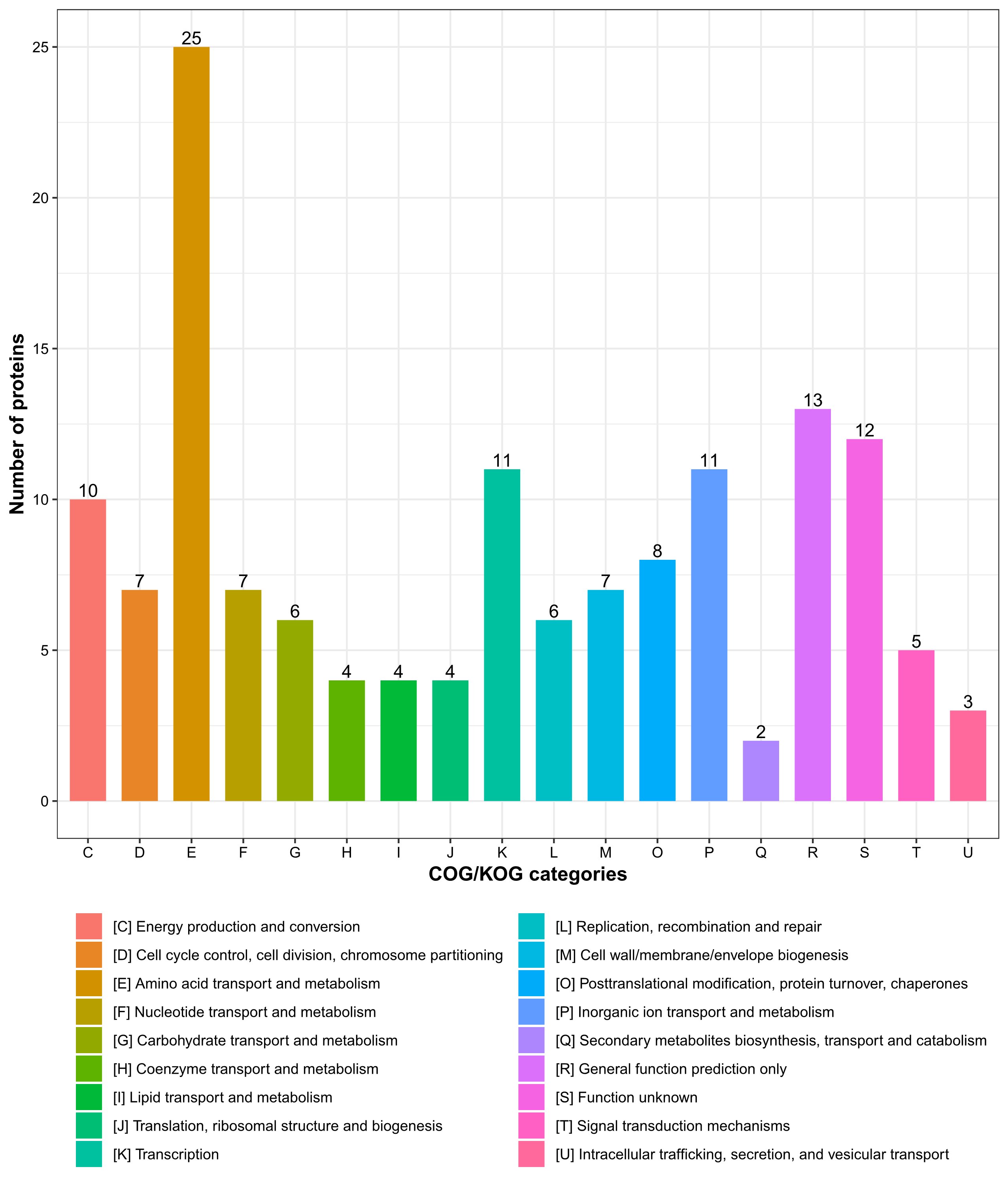

Supplement: Supplementary file 1 [file DataSheet_1.zip › Supplementary Figure 6.jpg]

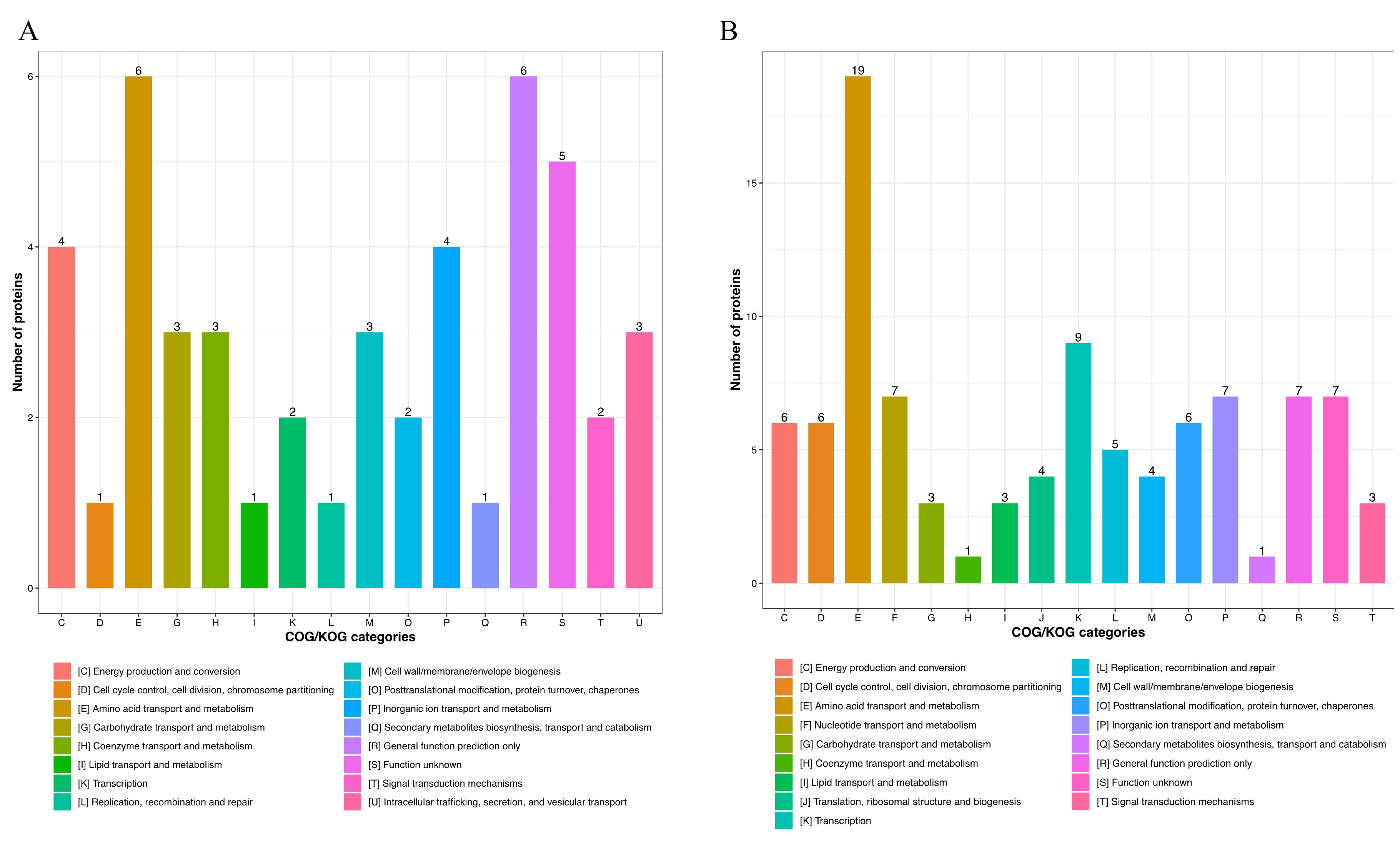

Supplement: Supplementary file 1 [file DataSheet_1.zip › Supplementary Figure 7.jpg]

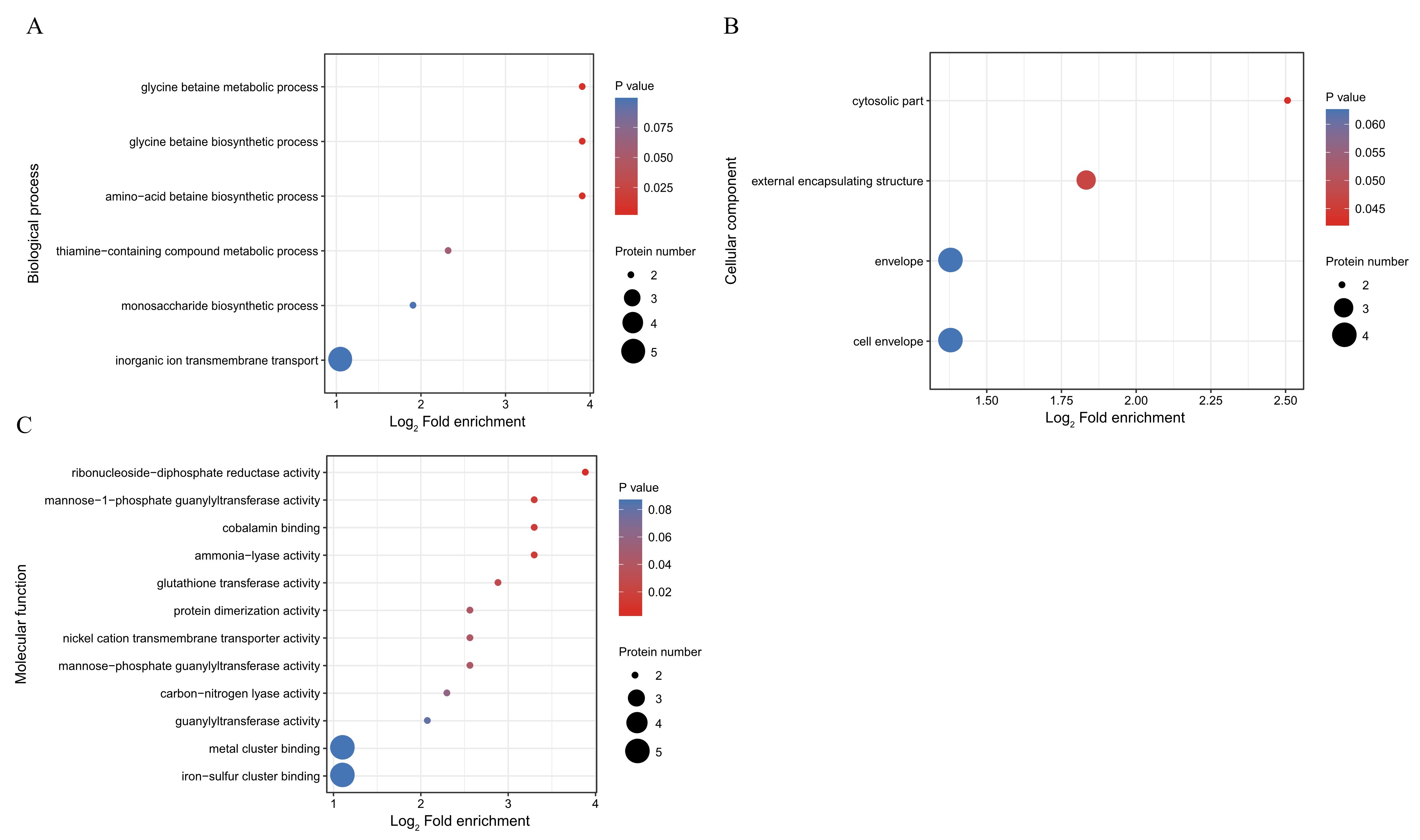

Supplement: Supplementary file 1 [file DataSheet_1.zip › Supplementary Figure 8.jpg]

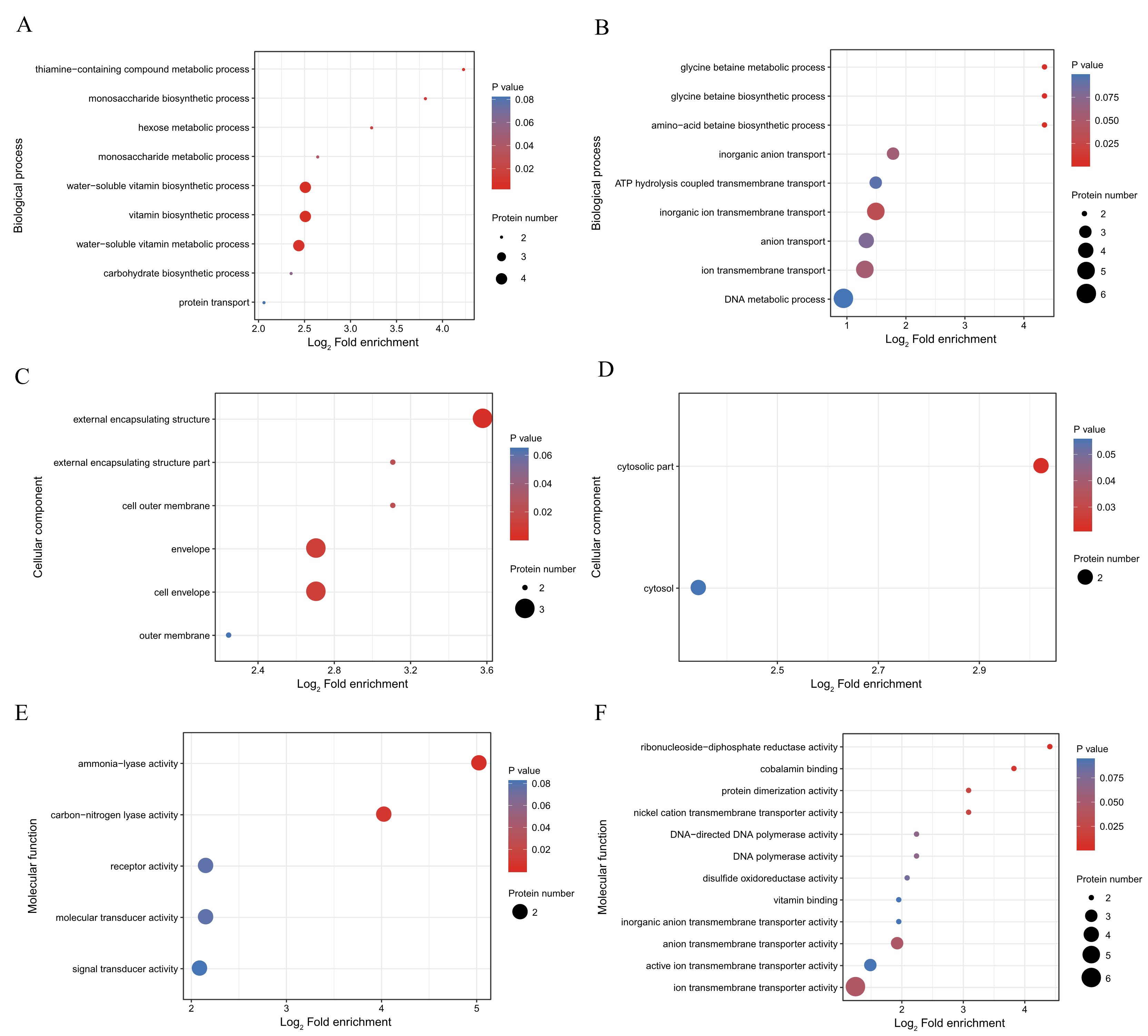

Supplement: Supplementary file 1 [file DataSheet_1.zip › Supplementary Figure 9.jpg]

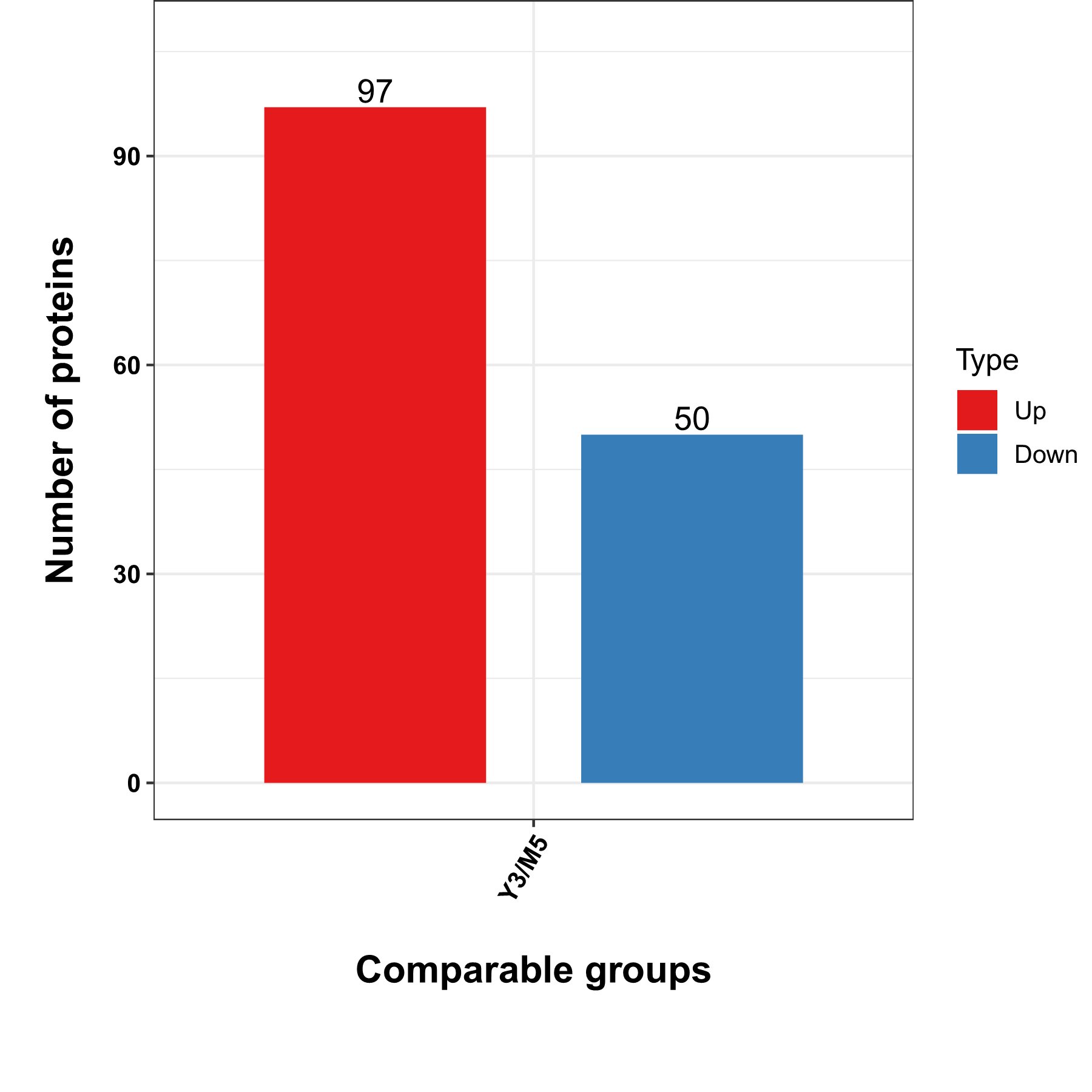

Supplement: Supplementary file 1 [file DataSheet_1.zip › Supplemtary Figure 2.jpg]
